# Two New Andrastin-Type Meroterpenoids from Marine Sponge-Derived Fungus *Botryosporium* sp. S5I2-1

**DOI:** 10.3390/molecules31020294

**Published:** 2026-01-14

**Authors:** Hui-Xian Liang, Wan-Ying Guo, Shi-Hai Xu, Bing-Xin Zhao

**Affiliations:** Department of Chemistry, College of Chemistry and Materials Science, Jinan University, Guangzhou 510632, China

**Keywords:** sponge-derived fungus, *Botryosporium*, andrastin-type meroterpenoids

## Abstract

Botryomeroterpenoids A (**1**) and B (**2**), two new andrastin-type meroterpenoids, along with two known analogues (**3** and **4**), were isolated from sponge-derived fungus *Botryosporium* sp. S5I2-1. Their structures were characterized by detailed spectroscopic analysis. Meanwhile, the absolute configurations of **1** and **2** were elucidated by comparing experimental and calculated ECD spectra. Compounds **1** and **2** were the first examples of andrastin-type meroterpenoids isolated from this genus, especially Compound **1** which represented the initial instance of 18-norandrastin-type meroterpenoids. Furthermore, the antibacterial activities of all compounds were also evaluated. However, the results indicated that these compounds showed no significant inhibitory activity against the tested bacteria with minimum inhibitory concentration (MIC) values of 32–64 μg/mL.

## 1. Introduction

Fungi generated from marine sponges are widely recognized as abundant sources of bioactive natural compounds, which are currently a valuable resource for drug discovery. Numerous secondary metabolites [1], mainly alkaloids [2], polyketides [3], terpenes [4] and sterols [5], etc., have been consistently isolated from marine sponge-derived fungus. A large proportion of these compounds display a wide range of biological activities [1], including antibacterial [6], cytotoxic [7], antiviral [8], etc. For example, 4-*O*-methylcandidusin A, which was isolated from marine sponge-derived fungus *Aspergillus candidus* OUCMDZ-1051, selectively inhibited the growth of the MDA-MB-468, BT474 and A431 cell lines with IC_50_ values of 1.84, 6.05, and 0.98 μmol/L, respectively, thereby indicating its potential as a lead compound for the discovery of novel drugs against these malignancies [9]. Another compound, amauromine, isolated from the sponge-derived fungus *Auxarthron reticulatum*, acted as an antagonist of the cannabinoid-like orphan receptor GPR18 (IC_50_ = 3.74 µM) and thus held promise as a lead structure for developing more potent and selective GPR18 antagonists [10]. Andrastin-type meroterpenoids, which are biosynthesized from 5-dimethylorsellinic acid (DMOA) and farnesyl diphosphate (FPP) via the mixed polyketide–terpenoid pathway, are defined by a 5-methyl-substituted ent-5α,14β-androstane scaffold corresponding to a 6,6,6,5-tetracarbocyclic framework. Except for a small portion of these compounds derived from the genus *Aspergillus*, more than seventy andrastin-type meroterpenoids have been identified and isolated from the genus *Penicillium*. Some of these compounds have shown several biological activities, including cytotoxic, anti-inflammatory, antifeedant, insecticidal, and antibacterial activity [11,12,13]. The fungus of genus *Botryosporium* belongs to the order Moniliales and the family Moniliaceae. This extensively dispersed genus of plant pathogenic fungus encompasses five identified species: *B. hughesii*, *B. longibrachiatum* var. *longibrachiatum*, *B. longibrachiatum* var. *macrosporum*, *B. madrasense*, and *B. pulchrum*. These fungi can infect various woody and herbaceous plants, with a higher prevalence in temperate and subtropical climatic regions [14,15,16,17]. Additionally, the morphological characteristics of the main taxonomic units of the genus *Botryosporium* have been studied [18,19]. Only *B. pulchrum* has been isolated from sponges, and this genus rarely occurs in marine habitats. Merely three secondary metabolites, all azaphilones, have been isolated from this genus in our research to date. These compounds exhibited potential anti-inflammatory activities in a CuSO_4_-induced transgenic zebrafish paradigm [20].

**Figure 1:**
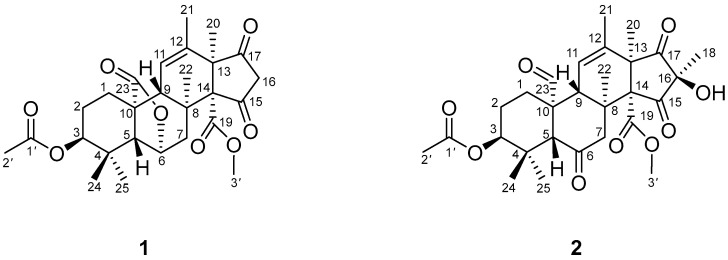
Chemical structures of **1** and **2**.

Structurally distinct secondary metabolites with biological activity from marine sponge-derived fungus are the focus of our ongoing investigations [21,22,23]. Thus, two new andrastin-type meroterpenoids, botryomeroterpenoids A (**1**) and B (**2**), along with two known ones (**3** and **4**), were isolated from marine sponge-derived fungus *Botryosporium* sp. S5I2-1 (Figure 1). Compounds **1** and **2** were the first examples of andrastin-type meroterpenoids isolated from this genus, especially Compound **1** which represented the initial instance of 18-norandrastin-type meroterpenoids.

Herein, the isolation, structural elucidation, and biological activities of these compounds are described.

## 2. Results and Discussion

Compound **1** was obtained as a yellow powder. The molecular formula of **1** was established as C_27_H_34_O_8_ from HR-ESI-MS (*m*/*z* 487.2331 [M + H]^+^, calcd for C_27_H_35_O_8_, 487.2326), indicating 11 degrees of unsaturation. The UV absorption maximum was at 202 nm. The IR bands indicated the presence of three carbonyl groups (1767, 1711, and 1619 cm^−1^). The ^1^H NMR (600 MHz, CD_3_OD) spectrum showed one olefinic proton [*δ*_H_ 5.59 (1H, br s)], four methines [*δ*_H_ 4.82 (1H, d, *J* = 4.4 Hz), 3.36 (1H, br s), 2.51 (1H, d, *J* = 4.4 Hz) and 2.07 (1H, s)], four methylenes [*δ*_H_ 3.35 (2H, s), 3.26 (1H, d, *J* = 10.5 Hz), 2.48 (1H, dd, *J* = 9.6, 4.4 Hz), 2.02 (2H, dd, *J* = 14.7, 5.8 Hz) and 1.72 (2H, dd, *J* = 14.9, 5.7 Hz)], and seven methyls [*δ*_H_ 3.59 (3H, s), 1.84 (3H, s), 1.60 (3H, s), 1.31 (3H, s), 1.24 (3H, s), 1.04 (3H, s) and 0.79 (3H, s)]. The ^13^C NMR (150 MHz, CD_3_OD) data revealed the presence of 27 carbons, including five carbonyl at *δ*_C_ 205.7, 193.2, 182.6, 172.5, and 166.5, a double-bond at *δ*_C_ 138.9 and 123.2, five quaternary carbons at *δ*_C_ 78.5, 71.2, 45.1, 43.3, and 36.2, four methines at *δ*_C_ 80.2, 74.4, 55.5, and 52.7, and four methylenes at *δ*_C_ 49.8, 38.2, 25.8, and 21.4, as well as seven methyls at *δ*_C_ 52.0, 26.9, 25.0, 24.3, 23.2, 20.5, and 17.8 (Table 1).

**Figure 2:**
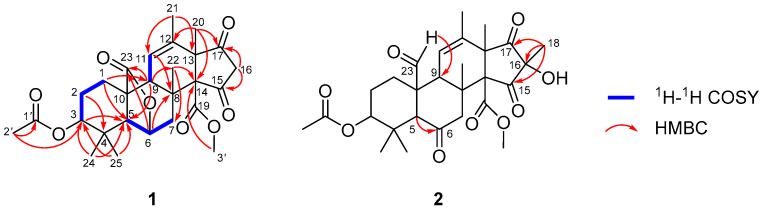
^1^H-^1^H-COSY and key HMBC correlations of **1** and **2**.

In the ^1^H-^1^H-COSY spectrum, three spin coupling systems were deduced as shown in Figure 2. Subsequently, based on the HMBC correlations from H-1 to C-5/C-9, from H-2 to C-4, from H-3 to C-5, from H-6 to C-8/C-23, from H-9 to C-5/C-23, from H-11 to C-8, from H-16 to C-15/C-17, from H-20 to C-12/C-14/C-17, from H-21 to C-11/C-13, from H-22 to C-7/C-14, the skeleton of pentacyclic meroterpenoid with 6/5/6/6/5 system was elucidated. According to the HMBC correlations between H-24/H-25 and C-3/C-5, the two methyl groups were linked to C-4. Meanwhile, the HMBC correlations between H-2′ and C-3/C-1′ implied that the acetoxyl was connected through C-3. Furthermore, the molecular formula information together with the obvious downfield chemical shifts in C-14, as well as the HMBC correlations from H-3′ to C-14, suggested that the methoxycarbonyl were attached at C-14. Hence, the planar structure of **1** was established, which was similar to that of the known Compound **3**. The only difference was the absence of one hydroxyl and one methyl group at the C-16 position in **1** [24].

**Figure 3:**
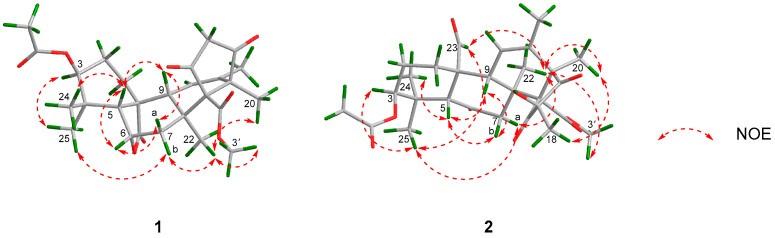
Key NOE correlations of **1** and **2**.

**Figure 4:**
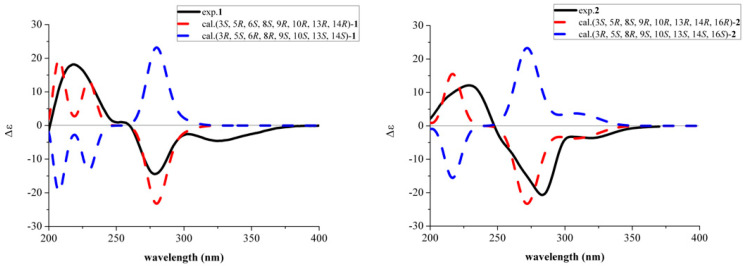
Experimental and calculated ECD spectra of **1** and **2**.

The relative configuration of **1** was determined by analysis of the NOESY spectrum. The correlations of H-5 and H-6/H-9/H-24, as well as H-7a and H-6/H-9, confirmed that these protons were cofacial, which were assigned as β-oriented. Accordingly, the NOE correlations between H-25 and H-3/H-7b together with H-22 and H-7b/H-20/H-3′ demonstrated that these protons were α-oriented (Figure 3). Hence, the relative configuration of **1** was completely established as 3S*, 5R*, 6S*, 8S*, 9R*, 10R*, 13R*, and 14R*. The absolute configuration of **1** was determined by the electronic circular dichroism (ECD) calculation. As a result, the calculated ECD spectrum of (3*S*, 5*R*, 6*S*, 8*S*, 9*R*, 10*R*, 13*R*, 14*R*)-**1** matched well with the experimental one (Figure 4), which allowed the absolute configuration of **1** as 3*S*, 5*R*, 6*S*, 8*S*, 9*R*, 10*R*, 13*R* and 14*R*.

Compound **2** was obtained as a yellow powder. The molecular formula of **2** was established as C_28_H_36_O_9_ from HR-ESI-MS (*m*/*z* 539.2244 [M + Na]^+^, calcd for C_28_H_36_O_9_Na, 539.2252), indicating 11 degrees of unsaturation. The UV absorption maximum was at 203 nm. The IR bands indicated the presence of the carbonyl groups (1731 and 1603 cm^−1^). The 1D NMR data of **2** (Table 1) were very similar to those of **1**, indicating that these two compounds might be the analogues. The differences between them were that **2** had one more carbonyl (*δ*_C_ 206.7), quaternary carbon (*δ*_C_ 72.8), and methyl [*δ*_H_ 1.31 (3H, s); *δ*_C_ 20.4] group, whereas the signals of a methine and a methylene had disappeared.

The HMBC correlations from H-18 to C-15/C-16/C-17 indicated that C-18 was attached to C-16. The HMBC correlation between H-23 to C-9 implied that the aldehyde group was connected to C-10. Meanwhile, the HMBC correlation from H-5 to C-6 confirmed the existence of a carbonyl group at C-6. The hydroxy group was assigned at C-16, which was determined by the obvious downfield chemical shifts in C-16 as well as the molecular formula information (Figure 2). The NOE correlations between H-5 and H-7b/H-9/H-24 showed that these protons were cofacial which were assigned as β-oriented. Therefore, the NOE correlations between H-25 and H-3/H-7a/H-23 as well as between H-22 and H-7a/H-20/H-23/H-3′ demonstrated that these protons were α-oriented (Figure 3). Hence, the relative configuration of **2** was completely established as 3S*, 5R*, 8S*, 9R*, 10R*, 13R*, 14R*, and 16R*. The absolute configuration of **2** was determined by the ECD calculation. As a result, the calculated ECD spectrum of (3*S*, 5*R*, 8*S*, 9*R*, 10*R*, 13*R*, 14*R*, 16*R*)-**2** matched well with the experimental one (Figure 4), which allowed the absolute configuration of **2** as 3*S*, 5*R*, 8*S*, 9*R*, 10*R*, 13*R*, 14*R*, and 16*R*. In summary, Compounds **1** and **2** exhibited similar Cotton effects to those of the known compounds [13,24,25], whose absolute configurations were also determined by means of ECD calculations.

The two known compounds were identified as 16-epi-citreohybriddione (**3**) and citreohybriddione (**4**) [24], which were compared with the reported literature. Given that previous studies had reported the antibacterial activity of andrastin-type meroterpenoids [26], we aimed to further identify analogues with more potent antibacterial activity. Thus, the antibacterial activities of **1**–**4** were evaluated against *Pseudomonas aeruginosa* ATCC27853, *Escherichia coli* ATCC25922, *Enterococcus faecalis* ATCC29212, *Staphylococcus aureus* ATCC29213, *Klebsiella pneumoniae* ATCC700603, and *Acinetobacter baumnnii* ATCC19606. Unfortunately, the results indicated that these compounds showed no significant inhibitory activity against the tested bacteria with minimum inhibitory concentration (MIC) values of 32–64 μg/mL. Therefore, we will further enrich a larger number of this type of compounds and evaluate their multiple biological activities, such as antibacterial, cytotoxic, and anti-inflammatory activities, to discuss their structure–activity relationships.

## 3. Materials and Methods

### 3.1. General Experimental Procedures

The P-2000 Digital Polarimeter (JASCO International Co., Ltd., Hachioji, Japan) was applied to measure optical rotations. The Shimadzu UV-2600 Plus spectrometer (Shimadzu, Kyoto, Japan) and Nicolet iS 50 FT-IR (Thermo, Waltham, MA, USA) were utilized to collect the UV and IR spectra, respectively. HR-ESI-MS was assessed using the Agilent 6210 LC/MSD TOF mass spectrometer (Agilent, Palo Alto, CA, USA). NMR spectra were recorded on Bruker av 600 NMR spectrometers (Bruker, Fällanden, Switzerland). Structural assignments were made with additional information from the gNOESY, gCOSY, gHSQC, and gHMBC experiments. ECD spectrometer (JASCO International Co., Ltd., Hachioji, Tokyo, Japan) served to collect CD spectra. An Agilent 1200 series device (Agilent, Palo Alto, CA, USA) was used to perform semi-preparative HPLC [Rp-C18: 20 × 250 mm i.d, 10 µm (Cosmosil, Nacalai, Kyoto, Japan)]. Silica gel (300–400 mesh, Qingdao Haiyang Chemical Co., Qingdao, China) and Se-phadex LH-20 (GE Healthcare, Uppsala, Sweden) were the materials employed for column chromatography. Precoated silica gel plates (GF-254, Jiangyou Silica Gel Development, Inc., Yantai, China) were applied for thin-layer chromatography.

### 3.2. Fungal Material

The fungal strain S5I2-1 was obtained from marine sponge *Gelliodes* sp., which was gathered from the maritime region of Xuwen County, Zhanjiang City, China. It was identified as *Botryosporium* sp. based on sequencing of the ITS region (GenBank no. MH854672.1) with 99% similarity, which was deposited in the Department of Chemistry, College of Chemistry and Materials Science, Jinan University.

### 3.3. Fermentation, Extraction, and Isolation

The fungal strain *Botryosporium* sp. S5I2-1 was grown on solid medium I2 (glucose 1.6 g, yeast extract 1.6 g, malt extract 4.0 g, agar 6.0 g, sea salt 14.0 g and 400 mL distilled water) at 28 °C for a week. The mycelium was carefully excised from the Petri dish using an inoculation loop and transferred to 500 mL conical flask pre-filled with 100 mL of sterile liquid medium I2. To create the seed culture that was utilized to inoculate the rice medium, the liquid culture was incubated for three days at 180 rpm and 28 °C in a rotatory shaker (50 × 500 mL conical flask; 70 g of rice; 3 g of sea salt; 110 mL of distilled water in each flask). The fermentation was carried out at 28 °C under static condition for 50 days. Then the cultures were soaked and extracted with EtOAc for three times, which was concentrated in vacuo to gain a crude extract (9.0 g). Next, the extract was suspended in 90% MeOH-H_2_O and partitioned with cyclohexane to obtain MeOH-H_2_O fraction (MF, 2.3 g) and cyclohexane fraction (6.4 g).

The MF was separated into six fractions (Frs. 1–6) by an open ODS chromatographic column (CC), eluting with MeOH/H_2_O in gradient eluent (10:90~100:0). Gradient elution of Fr. 4 (624.8 mg) on silica gel column chromatography (CC) with dichloromethane/methanol (100:0 to 0:100) afforded six sub-fractions (Frs. 4.1–4.6). Four sub-fractions (Frs. 5.1–5.4) were isolated from Fr. 5 (407.7 mg) by silica gel CC using a dichloromethane/methanol gradient (100:0 to 0:100).

Semi-preparative HPLC with a mobile phase of MeOH/H_2_O (75:25, *v*/*v*) was applied to separate Fr. 4.3 (32.4 mg). Compounds **3** (1.5 mg, 2.5 mL/min, t_R_: 31.5 min) and **4** (1.2 mg, 2.5 mL/min, t_R_: 35.3 min) were afforded. Compound **1** (0.5 mg) was obtained from Fr. 4.4 (27.5 mg) through semi-preparative HPLC [MeOH/H_2_O (75:25, *v*/*v*), 2.5 mL/min, t_R_: 30.2 min]. Using semi-preparative HPLC [MeOH/H_2_O 75:25 (*v*/*v*), 2.5 mL/min, t_R_: 40.5 min], **2** (0.8 mg) was obtained from Fr. 5.2 (34.4 mg).

### 3.4. Antibacterial Activities

The broth microdilution method was used to evaluate antibacterial activity against a variety of bacterial strains, including *Pseudomonas aeruginosa* ATCC27853, *Escherichia coli* ATCC25922, *Enterococcus faecalis* ATCC29212, *Staphylococcus aureus* ATCC29213, *Klebsiella pneumoniae* ATCC700603, and *Acinetobacter baumnnii* ATCC19606. Ciprofloxacin (J&K Chemical Technology, Beijing, China) was utilized as a positive control in each experiment, which was carried out in triplicate.

### 3.5. ECD Calculations

The molecules of (3*S*, 5*R*, 6*S*, 8*S*, 9*R*, 10*R*, 13*R*, 14*R*)-**1** and (3*R*, 5*S*, 6*R*, 8*R*, 9*S*, 10*S*, 13*S*, 14*S*)-**1** (Figure S1) were converted into SMILES codes before their initial 3D structures were generated with CORINA version 3.4. Conformer databases were generated in CONFLEX version 7.0 using the MMFF94s force field, with an energy window for acceptable conformers (ewindow) of 5 kcal/mol above the ground state, a maximum number of conformations per molecule (maxconfs) of 100, and an RMSD cutoff (rmsd) of 0.5 Å. Then each acceptable conformer was optimized with the HF/6-31G(d) method in Gaussian09. Further optimization at the wB97XD/6-31G(d) level determined the dihedral angles. From this, two stable conformers were obtained for each molecule (Figure S2). The optimized conformer was used for the ECD calculation, which was performed with Gaussian09 (wB97XD/TZVP). The solvent effect was accounted by the polarizable-conductor calculation model (IEFPCM, methanol as the solvent). Comparisons of the experimental and calculated spectra were performed with the SpecDis software (version 1.71, University of Potsdam, Potsdam, Germany). This was also used to apply a UV shift to the ECD spectra, Gaussian broadening of the excitations, and Boltzmann weighting of the spectra (Table S1).

The molecules of (3*S*, 5*R*, 8*S*, 9*R*, 10*R*, 13*R*, 14*R*, 16*R*)-**2** and (3*R*, 5*S*, 8*R*, 9*S*, 10*S*, 13*S*, 14*S*, 16*S*)-**2** (Figure S3) were converted into SMILES codes before their initial 3D structures were generated with CORINA version 3.4. Conformer databases were generated in CONFLEX version 7.0 using the MMFF94s force field, with an energy window for acceptable conformers (ewindow) of 5 kcal/mol above the ground state, a maximum number of conformations per molecule (maxconfs) of 100, and an RMSD cutoff (rmsd) of 0.5 Å. Then each acceptable conformer was optimized with the HF/6-31G(d) method in Gaussian09. Further optimization at the wB97XD/6-31G(d) level determined the dihedral angles. From this, six stable conformers were obtained for each molecule (Figure S4). The optimized conformer was used for the ECD calculation, which was performed with Gaussian09 (wB97XD/TZVP). The solvent effect was accounted by the polarizable-conductor calculation model (IEFPCM, methanol as the solvent). Comparisons of the experimental and calculated spectra were performed with the SpecDis software (version 1.71, University of Potsdam, Potsdam, Germany). This was also used to apply a UV shift to the ECD spectra, Gaussian broadening of the excitations, and Boltzmann weighting of the spectra (Table S3).

## 4. Conclusions

In summary, botryomeroterpenoids A (**1**) and B (**2**), two new andrastin-type meroterpenoids, along with two known compounds (**3** and **4**), were isolated from sponge-derived fungus *Botryosporium* sp. S5I2-1. Compound **1** uniquely exemplified the first instance of 18-norandrastin-type meroterpenoids. Both of **1** and **2** were the first cases of andrastin-type meroterpenoids isolated from this genus. However, all the compounds showed no significant inhibitory activities against the tested bacteria.

## Figures and Tables

**Table 1:** ^1^H (600 MHz) and ^13^C NMR (150 MHz) spectroscopic data of **1** and **2** in CD_3_OD (*J* in Hz).

NO.	1		2	
	*δ*_H_	*δ*_C_	*δ*_H_	*δ*_C_
1	2.02 dd (14.7, 5.8)	21.4	1.57 m	24.3
2	1.72 dd (14.9, 5.7)	25.8	2.36 m	28.5
3	3.36 br s	74.4	4.62 t (5.6)	79.0
4	-	36.2	-	38.0
5	2.07 s	55.5	2.26 s	54.8
6	4.82 d (4.4)	80.2	-	206.7
7	a 2.48 dd (6.9, 4.4)	38.2	a 2.36 m	32.2
	b 3.26 d (14.2)		b 2.88 td (12.9, 3.8)	
8	-	43.3	-	40.1
9	2.51 d (4.4)	52.7	1.81 d (3.5)	49.4
10	-	45.1	-	53.5
11	5.59 br s	123.2	5.67 br s	126.6
12	-	138.9	-	133.0
13	-	71.2	-	61.5
14	-	78.5	-	72.1
15	-	193.2	-	212.1
16	3.35 s	49.8	-	72.8
17	-	205.7	-	209.3
18			1.31 s	20.4
19	-	166.5	-	169.2
20	1.24 s	17.8	1.27 s	17.3
21	1.84 s	20.5	1.63 s	19.0
22	1.31 s	24.3	1.19 s	20.2
23	-	182.6	10.18 s	206.7
24	1.04 s	26.9	0.90 s	21.6
25	0.79 s	23.2	0.97 s	27.1
1′	-	172.5	-	172.3
2′	1.60 s	25.0	2.06 s	21.1
3′	3.59 s	52.0	3.60 s	52.2

## Data Availability

All relevant data are available in the article and its Supplementary Materials.

## Supplementary Materials

The following supporting information can be downloaded at https://www.mdpi.com/article/10.3390/molecules31020294/s1, compound characterization data; Figure S1. (3*S*, 5*R*, 6*S*, 8*S*, 9*R*, 10*R*, 13*R*, 14*R*)-**1** and (3*R*, 5*S*, 6*R*, 8*R*, 9*S*, 10*S*, 13*S*, 14*S*)-**1**; Figure S2. Stable conformers of (3*S**, 5*R**, 6*S**, 8*S**, 9*R**, 10*R**, 13*R**, 14*R**)-**1** at the wB97XD/6-31G(d) level in methanol; Figure S3. (3*S*, 5*R*, 8*S*, 9*R*, 10*R*, 13*R*, 14*R*, 16*R*)-**2** and (3*R*, 5*S*, 8*R*, 9*S*, 10*S*, 13*S*, 14*S*, 16*S*)-**2**; Figure S4. Stable conformers of (3*S**, 5*R**, 8*S**, 9*R**, 10*R**, 13*R**, 14*R**, 16*R**)-**2** at the wB97XD/6-31G(d) level in methanol; Figure S5. HRESIMS spectrum of **1**; Figure S6. UV spectrum of **1** (MeOH); Figure S7. IR spectrum of **1** (MeOH); Figure S8. ^1^H NMR spectrum (600 MHz, CD_3_OD) of **1**; Figure S9. ^13^C NMR spectrum (150 MHz, CD_3_OD) of **1**; Figure S10. ^1^H-^1^H-COSY spectrum of **1**; Figure S11. HSQC spectrum of **1**; Figure S12. HMBC spectrum of **1**; Figure S13. NOESY spectrum of **1**; Figure S14. HRESIMS spectrum of **2**; Figure S15. UV spectrum of **2** (MeOH); Figure S16. IR spectrum of **2** (MeOH); Figure S17. ^1^H NMR spectrum (600 MHz, CD_3_OD) of **2**; Figure S18. ^13^C NMR spectrum (150 MHz, CD_3_OD) of **2**; Figure S19. ^1^H-^1^H-COSY spectrum of **2**; Figure S20. HSQC spectrum of **2**; Figure S21. HMBC spectrum of **2**; Figure S22. NOESY spectrum of **2**; Table S1. The energies and Boltzmann distributions for stable conformers of (3*S**, 5*R**, 6*S**, 8*S**, 9*R**, 10*R**, 13*R**, 14*R**)-**1**; Table S2. Cartesian coordinates of all conformers of Compound **1**; Table S3. The energies and Boltzmann distributions for stable conformers of (3*S**, 5*R**, 8*S**, 9*R**, 10*R**, 13*R**, 14*R**, 16*R**)-**2**; Table S4. Cartesian coordinates of all conformers of Compound **2**.
