# Supplementary material for: Two New Andrastin-Type Meroterpenoids from Marine Sponge-Derived Fungus Botryosporium sp. S5I2-1
Source: Molecules. 2026 Jan 14;31(2):294. doi: 10.3390/molecules31020294 (PMC12843713; doi:10.3390/molecules31020294)
Supplement: Supplementary file 1 [file molecules-31-00294-s001.zip › molecules-4064241-supplementary.pdf]

Supporting Information for

Two New Andrastin-Type Meroterpenoids from Marine Sponge-Derived Fungus *Botryosporium* sp. S5I2-1

Hui-Xian Liang, Wan-Ying Guo, Shi-Hai Xu* and Bing-Xin Zhao*

Department of Chemistry, College of Chemistry and Materials Science, Jinan University, Guangzhou, 510632, China

* Corresponding authors: txush@jnu.edu.cn (S.-H.X.); zbx840622@163.com (B.-X.Z.)

TABLE OF CONTENTS

1. **Compound characterization data** 3
2. **Quantum chemical ECD calculations of 1** 3
    Figure S1. (3*S*, 5*R*, 6*S*, 8*S*, 9*R*, 10*R*, 13*R*, 14*R*)-**1** and (3*R*, 5*S*, 6*R*, 8*R*, 9*S*, 10*S*, 13*S*, 14*S*)-**1** .. 3
    Figure S2. Stable conformers of (3*S**, 5*R**, 6*S**, 8*S**, 9*R**, 10*R**, 13*R**, 14*R**)-**1** at the wB97XD/6-31G(d) level in methanol.. 3
    Table S1. The energies and boltzmann distributions for stable conformers of (3*S**, 5*R**, 6*S**, 8*S**, 9*R**, 10*R**, 13*R**, 14*R**)-**1** 4
    Table S2. Cartesian coordinates of all conformers of compound **1**.. 4
3. **Quantum chemical ECD calculations of 2** 8
    Figure S3. (3*S*, 5*R*, 8*S*, 9*R*, 10*R*, 13*R*, 14*R*, 16*R*)-**2** and (3*R*, 5*S*, 8*R*, 9*S*, 10*S*, 13*S*, 14*S*, 16*S*)-**2** 8
    Figure S4. Stable conformers of (3*S**, 5*R**, 8*S**, 9*R**, 10*R**, 13*R**, 14*R**, 16*R**)-**2** at the wB97XD/6-31G(d) level in methanol.. 8
    Table S3. The energies and boltzmann distributions for stable conformers of (3*S**, 5*R**, 8*S**, 9*R**, 10*R**, 13*R**, 14*R**, 16*R**)-**2**.. 9
    Table S4. Cartesian coordinates of all conformers of compound **2**.. 9
4. **Spectra of 1** 22
    Figure S5. HRESIMS spectrum of **1**.. 23
    Figure S6. UV spectrum of **1** (MeOH).. 23
    Figure S7. IR spectrum of **1** (MeOH). 24
    Figure S8. ^{1}H NMR spectrum (600 MHz, CD_3OD) of **1**. 24
    Figure S9. ^{13}C NMR spectrum (150 MHz, CD_3OD) of **1**. 24
    Figure S10. ^{1}H–^{1}H COSY spectrum of **1**. 25
    Figure S11. HSQC spectrum of **1**.. 25
    Figure S12. HMBC spectrum of **1**. 26
    Figure S13. NOESY spectrum of **1**.. 26
5. **Spectra of 2** 27
    Figure S14. HRESIMS spectrum of **2**.. 27
    Figure S15. UV spectrum of **2** (MeOH).. 27
    Figure S16. IR spectrum of **2** (MeOH). 27
    Figure S17. ^{1}H NMR spectrum (600 MHz, CD_3OD) of **2**. 28
    Figure S18. ^{13}C NMR spectrum (150 MHz, CD_3OD) of **2**. 28
    Figure S19. ^{1}H–^{1}H COSY spectrum of **2**. 29
    Figure S20. HSQC spectrum of **2**. 29
    Figure S21. HMBC spectrum of **2**. 30
    Figure S22. NOESY spectrum of **2**.. 30

1. Compound characterization data

Botryomeroterpenoid A (1): Yellow powders; $[\alpha]_D^{25}$ -67.0 (*c* 0.10 CH_3OH); UV (MeOH) λ_{max} (log ε) 202 nm; ECD (MeOH) λ_{max} ($\Delta\varepsilon$) 219 (+18.15), 280 (-14.27) nm; IR (MeOH) ν_{max} 3421, 2964, 2877, 1767, 1711, 1619, 1450, 1399, 1260, 1205, 1126, 1095, 1032, 934 and 798 cm^{-1}; HR-ESI-MS *m/z* 487.2331 $[M + H]^+$ (calcd for $C_{27}H_{35}O_8$, 487.2326); ^1H and ^{13}C NMR data see Table S1.

IUPAC name: (3*S*,5*R*,6*S*,8*S*,9*R*,10*R*,13*R*,14*R*)-3-acetoxy-20,21,22,24,25-pentamethyl-15,17,23-trioxo-dodecahydro-6,10-(epoxymethano) cyclopenta [a] phenanthrene-14-carboxylate

SMILES:
CC1(C)[C@@H](OC(C)=O)CC[C@]23[C@]1([H])[C@@H](OC3=O)C[C@@]4(C)[C@]([H])2C=C(C)[C@]5(C)[C@@]4(C(OC)=O)C(CC5=O)=O

Botryomeroterpenoid B (2): Yellow powders; $[\alpha]_D^{25}$ -50.0 (c 0.16 CH_3OH); UV (MeOH) λ_{max} (log ε) 203 nm; ECD (MeOH) λ_{max} ($\Delta\varepsilon$) 227 (+12.06), 283 (-20.69) nm; IR (MeOH) ν_{max} 3425, 2956, 2875, 1731, 1603, 1438, 1378, 1243, 1202 and 1034 cm^{-1}; HR-ESI-MS *m/z* 539.2244 $[M + Na]^+$ (calcd for $C_{28}H_{36}O_9Na$, 539.2252); ^1H and ^{13}C NMR data see Table S1.

IUPAC name: (3*S*,5*R*,8*S*,9*R*,10*R*,13*R*,14*R*,16*R*)-3-acetoxy-10-formyl-16-hydroxy-18,20,21,22,24,25-hexamethyl-6,15,17-trioxo-tetradecahydro-cyclopenta [a] phenanthrene-14-carboxylate

SMILES:
CC1(C)[C@@H](OC(C)=O)CC[C@]2(C=O)[C@]1([H])C(C[C@@]3(C)[C@@]2([H])C=C(C)[C@]4(C)[C@@]3(C(OC)=O)C([C@](C)(O)C4=O)=O)=O

2. Quantum chemical ECD calculations of 1

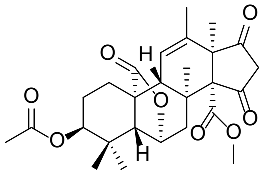

(3*S*, 5*R*, 6*S*, 8*S*, 9*R*, 10*R*, 13*R*, 14*R*)-**1**

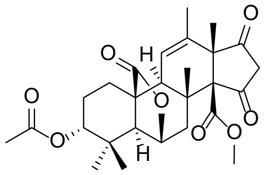

(3*R*, 5*S*, 6*R*, 8*R*, 9*S*, 10*S*, 13*S*, 14*S*)-**1**

Figure S1. (3*S*, 5*R*, 6*S*, 8*S*, 9*R*, 10*R*, 13*R*, 14*R*)-**1** and (3*R*, 5*S*, 6*R*, 8*R*, 9*S*, 10*S*, 13*S*, 14*S*)-**1**

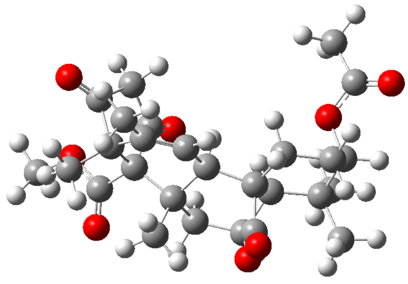
1-c1

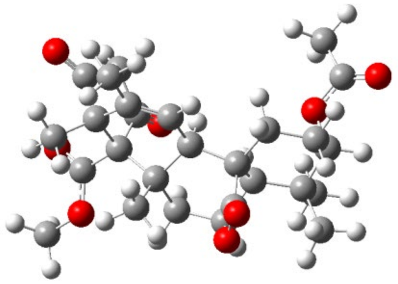
1-c2

Figure S2. Stable conformers of (3*S**, 5*R**, 6*S**, 8*S**, 9*R**, 10*R**, 13*R**, 14*R**)-**1** at the wB97XD/6-31G(d) level in methanol.

Table S1. The energies and Boltzmann distributions for stable conformers of (3*S**, 5*R**, 6*S**, 8*S**, 9*R**, 10*R**, 13*R**, 14*R**)-**1**

Conformations	Energies (Hartree)	Boltzmann distributions (%)	Imaginary frequency
**1**-c1	-1035854.21784187	94.2	0
**1**-c2	-1035852.56805533	5.8	0

Table S2. Cartesian coordinates of all conformers of compound **1**

conformation **1**-1c					
Center	Atomic	Atomic	Coordinates (Angstroms)		
Number	Number	Type	X	Y	Z
1	6	C	1.378452	-0.627276	0.852969
2	6	C	1.853578	-0.835936	-0.605197
3	6	C	1.031983	-2.08622	-0.922347
4	6	C	-0.435405	-1.74609	-1.184567
5	6	C	-1.104698	-1.047674	0.032261
6	6	C	-0.086569	-0.087037	0.732595
7	6	C	2.246295	0.298119	1.706227
8	6	C	3.7458	0.136672	1.45394
9	6	C	4.077203	0.163843	-0.03435
10	6	C	3.384855	-0.960719	-0.825854
11	6	C	-2.280833	-0.096956	-0.364211
12	6	C	-2.994399	0.553452	0.890865
13	6	C	-2.015058	0.782575	2.058181
14	6	C	-0.713884	0.490203	1.970823
15	6	C	-1.809763	1.129549	-1.214485
16	6	C	-2.357545	2.412977	-0.64469
17	6	C	-3.349419	1.974454	0.404296
18	6	C	3.99228	-2.304669	-0.380083
19	6	C	3.675733	-0.794742	-2.325521
20	8	O	3.613685	1.41602	-0.588459
21	6	C	4.420388	2.482305	-0.499458
22	6	C	3.775141	3.692268	-1.118904
23	8	O	5.519331	2.456152	0.011242
24	8	O	1.167143	-2.887066	0.287843
25	6	C	-1.648259	-2.154027	0.973621

26	6	C	-4.261848	-0.160497	1.383671
27	6	C	-3.334631	-0.756758	-1.260191
28	8	O	-3.43498	-1.926707	-1.538257
29	8	O	-4.267983	2.63769	0.824892
30	8	O	-1.130063	1.052248	-2.211644
31	6	C	-2.597998	1.349543	3.327796
32	1	H	0.070972	0.759255	0.051724
33	1	H	1.513036	-0.001571	-1.224613
34	8	O	-4.163949	0.186305	-1.727745
35	6	C	-5.232185	-0.259294	-2.575173
36	6	C	1.360724	-2.067653	1.342037
37	8	O	1.481673	-2.486834	2.467311
38	1	H	1.433762	-2.696015	-1.732945
39	1	H	-0.990253	-2.655516	-1.425337
40	1	H	-0.459148	-1.105919	-2.068832
41	1	H	2.038759	0.119228	2.76538
42	1	H	1.959192	1.333415	1.490431
43	1	H	4.11075	-0.801068	1.883881
44	1	H	4.291059	0.940944	1.956243
45	1	H	5.159035	0.106888	-0.184797
46	1	H	-0.083344	0.689917	2.832248
47	1	H	-1.542462	2.969813	-0.162552
48	1	H	-2.794453	3.054788	-1.411958
49	1	H	3.583689	-3.129572	-0.970679
50	1	H	5.07507	-2.285679	-0.545489
51	1	H	3.824643	-2.541362	0.672873
52	1	H	3.197191	-1.593475	-2.902681
53	1	H	4.753857	-0.848901	-2.514331
54	1	H	3.310305	0.164459	-2.701422
55	1	H	2.830818	3.908756	-0.611294
56	1	H	4.443368	4.549241	-1.038899
57	1	H	3.54787	3.49454	-2.170288
58	1	H	-0.943246	-2.978832	1.060781
59	1	H	-2.563999	-2.56959	0.549941

60	1	H	-1.86118	-1.785438	1.977314
61	1	H	-4.961492	-0.34315	0.564885
62	1	H	-4.779663	0.473887	2.105151
63	1	H	-4.025788	-1.111733	1.863158
64	1	H	-1.800586	1.655894	4.009557
65	1	H	-3.237097	2.216636	3.131473
66	1	H	-3.214467	0.605805	3.845348
67	1	H	-5.874142	-0.956166	-2.032861
68	1	H	-5.783253	0.6396	-2.84333
69	1	H	-4.827275	-0.744012	-3.465043
conformation **1**-2c					
Center	Atomic	Atomic	Coordinates (Angstroms)		
Number	Number	Type	X	Y	Z
1	6	C	1.30981	-0.442356	0.963571
2	6	C	1.795022	-0.95127	-0.415456
3	6	C	0.914088	-2.197812	-0.506034
4	6	C	-0.531069	-1.845799	-0.862233
5	6	C	-1.182256	-0.894028	0.180993
6	6	C	-0.122978	0.135607	0.703465
7	6	C	2.212344	0.587006	1.644197
8	6	C	3.704736	0.306752	1.464468
9	6	C	4.058237	0.032072	0.006672
10	6	C	3.320806	-1.189728	-0.571167
11	6	C	-2.301663	0.021116	-0.413972
12	6	C	-3.001639	0.925279	0.680408
13	6	C	-2.018571	1.351518	1.785839
14	6	C	-0.733916	0.982843	1.784868
15	6	C	-1.754865	1.054097	-1.461511
16	6	C	-2.311051	2.427645	-1.189817
17	6	C	-3.322316	2.224654	-0.089045
18	6	C	3.852124	-2.449741	0.138711
19	6	C	3.641509	-1.329422	-2.067384
20	8	O	3.66648	1.174581	-0.787783
21	6	C	4.521809	2.202007	-0.879543

22	6	C	3.946726	3.300392	-1.732134
23	8	O	5.608133	2.225386	-0.342467
24	8	O	0.990408	-2.755806	0.838003
25	6	C	-1.78239	-1.765603	1.316107
26	6	C	-4.289849	0.360146	1.297775
27	6	C	-3.411182	-0.63784	-1.237355
28	8	O	-4.184442	0.042518	-1.885417
29	8	O	-4.229938	2.976869	0.17766
30	8	O	-1.022398	0.775345	-2.381584
31	6	C	-2.580266	2.198936	2.899328
32	1	H	0.091695	0.819534	-0.126195
33	1	H	1.507188	-0.237115	-1.191842
34	8	O	-3.457204	-1.958315	-1.192911
35	6	C	-4.496301	-2.582519	-1.964271
36	6	C	1.210706	-1.758486	1.719736
37	8	O	1.293685	-1.958375	2.907054
38	1	H	1.294605	-2.971237	-1.174891
39	1	H	-1.123841	-2.76034	-0.933998
40	1	H	-0.511876	-1.389339	-1.854189
41	1	H	1.979348	0.626716	2.712429
42	1	H	1.983853	1.57376	1.226123
43	1	H	4.013384	-0.548044	2.074014
44	1	H	4.28437	1.164695	1.817161
45	1	H	5.137871	-0.103341	-0.104759
46	1	H	-0.100674	1.328478	2.596705
47	1	H	-1.505753	3.083235	-0.832114
48	1	H	-2.738722	2.881244	-2.086096
49	1	H	3.419537	-3.353371	-0.300048
50	1	H	4.93826	-2.508753	0.008044
51	1	H	3.647707	-2.47538	1.211252
52	1	H	3.126678	-2.197382	-2.493778
53	1	H	4.717411	-1.475313	-2.216081
54	1	H	3.335998	-0.441666	-2.62732
55	1	H	3.013399	3.660692	-1.289866

56	1	H	4.660107	4.120499	-1.808732
57	1	H	3.713501	2.914823	-2.728585
58	1	H	-1.133643	-2.607797	1.549887
59	1	H	-2.735352	-2.187499	0.993989
60	1	H	-1.945909	-1.200398	2.234155
61	1	H	-4.982305	0.013403	0.527301
62	1	H	-4.801723	1.150267	1.850223
63	1	H	-4.080659	-0.463216	1.982239
64	1	H	-1.773274	2.599359	3.518182
65	1	H	-3.170411	3.037623	2.515805
66	1	H	-3.240336	1.611525	3.547645
67	1	H	-4.383309	-2.328767	-3.019356
68	1	H	-4.366161	-3.651205	-1.808174
69	1	H	-5.474396	-2.256108	-1.606875

3. Quantum chemical ECD calculations of 2

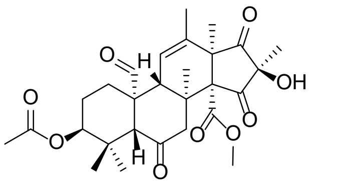

(3*S*, 5*R*, 8*S*, 9*R*, 10*R*, 13*R*, 14*R*, 16*R*)-**2**

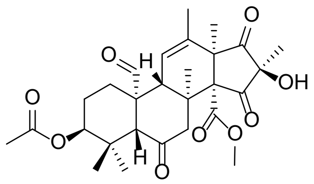

(3*R*, 5*S*, 8*R*, 9*S*, 10*S*, 13*S*, 14*S*, 16*S*)-**2**

Figure S3. (3*S*, 5*R*, 8*S*, 9*R*, 10*R*, 13*R*, 14*R*, 16*R*)-**2** and (3*R*, 5*S*, 8*R*, 9*S*, 10*S*, 13*S*, 14*S*, 16*S*)-**2**

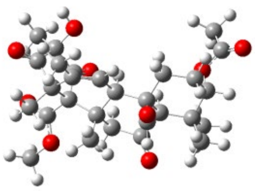

2-c1

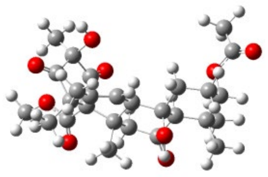

2-c2

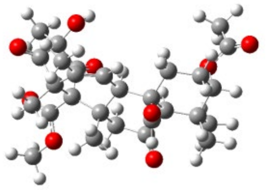

2-c3

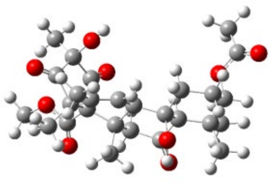

2-c4

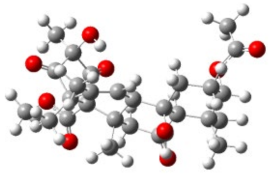

2-c5

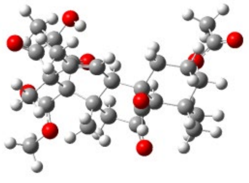

2-c6

Figure S4. Stable conformers of (3*S**, 5*R**, 8*S**, 9*R**, 10*R**, 13*R**, 14*R**, 16*R**)-**2** at the wB97XD/6-31G(d) level in methanol.

Table S3. The energies and Boltzmann distributions for stable conformers of (3*S**, 5*R**, 8*S**, 9*R**, 10*R**, 13*R**, 14*R**, 16*R**)-**2**

Conformations	Energies (Hartree)	Boltzmann distributions (%)	Imaginary frequency
**2**-c1	-1107678.35073258	1.43	0
**2**-c2	-1107679.84357887	17.78	0
**2**-c3	-1107678.58353879	2.12	0
**2**-c4	-1107680.08491921	26.73	0
**2**-c5	-1107680.43073997	47.95	0
**2**-c6	-1107678.95922902	3.99	0

Table S4. Cartesian coordinates of all conformers of compound **2**

conformation **2**-1c					
Center	Atomic	Atomic	Coordinates (Angstroms)		
Number	Number	Type	X	Y	Z
1	6	C	1.467226	-0.337252	1.028842
2	6	C	2.096878	-0.91806	-0.278873
3	6	C	1.225361	-2.049952	-0.803389
4	6	C	-0.181806	-1.629655	-1.169703
5	6	C	-0.908455	-1.031456	0.061524
6	6	C	-0.016852	0.06871	0.710259
7	6	C	2.250259	0.930427	1.432484
8	6	C	3.759338	0.703945	1.506077
9	6	C	4.314719	0.127202	0.209138
10	6	C	3.626037	-1.181859	-0.228914
11	6	C	-2.229659	-0.291101	-0.334526
12	6	C	-3.024042	0.277493	0.905537
13	6	C	-2.064608	0.781787	1.991363
14	6	C	-0.737971	0.686936	1.876943
15	6	C	-1.98436	0.975414	-1.22364
16	6	C	-2.868673	2.145411	-0.788399
17	6	C	-3.703862	1.538857	0.339671
18	6	C	4.052412	-2.294126	0.745777
19	6	C	4.127939	-1.562259	-1.632727
20	8	O	4.10561	1.09187	-0.847116

21	6	C	5.031064	2.045183	-1.022154
22	6	C	4.652356	2.954936	-2.159123
23	8	O	6.03253	2.145056	-0.346703
24	6	C	-1.199192	-2.189993	1.042873
25	6	C	-4.084471	-0.653769	1.511625
26	6	C	-3.246218	-1.054024	-1.191053
27	8	O	-4.163668	-0.460826	-1.727346
28	8	O	-4.721258	2.031272	0.768825
29	8	O	-1.213929	1.04157	-2.149118
30	6	C	-2.692049	1.38596	3.221574
31	6	C	1.492424	-1.340126	2.176311
32	6	C	-3.689767	2.731395	-1.921821
33	8	O	-1.970672	3.095526	-0.228716
34	8	O	1.594642	-3.203997	-0.91941
35	8	O	-3.071822	-2.359717	-1.279722
36	8	O	1.496387	-1.016194	3.344033
37	1	H	0.122759	0.850785	-0.047877
38	1	H	1.962076	-0.110981	-1.013514
39	6	C	-4.039507	-3.078911	-2.06381
40	1	H	-0.715929	-2.504317	-1.541518
41	1	H	-0.121068	-0.885021	-1.96847
42	1	H	1.901936	1.293434	2.40107
43	1	H	2.037258	1.712167	0.694568
44	1	H	4.006143	0.033102	2.335226
45	1	H	4.265022	1.650958	1.716662
46	1	H	5.3911	-0.04439	0.299156
47	1	H	-0.135525	1.069833	2.694573
48	1	H	3.555654	-3.230828	0.487908
49	1	H	5.134933	-2.446716	0.671532
50	1	H	3.835395	-2.066609	1.793261
51	1	H	3.77584	-2.558629	-1.907621
52	1	H	5.223893	-1.57054	-1.64807
53	1	H	3.78593	-0.850012	-2.388868
54	1	H	3.692062	3.43313	-1.945717

55	1	H	5.421365	3.714736	-2.295793
56	1	H	4.535556	2.373332	-3.077883
57	1	H	-0.378499	-2.911632	1.036095
58	1	H	-2.089367	-2.742775	0.743253
59	1	H	-1.335795	-1.839462	2.068148
60	1	H	-4.749018	-1.058899	0.744434
61	1	H	-4.709372	-0.084859	2.202567
62	1	H	-3.627221	-1.480424	2.057483
63	1	H	-1.929798	1.861085	3.843982
64	1	H	-3.44775	2.136591	2.968577
65	1	H	-3.190271	0.620555	3.827217
66	1	H	1.491438	-2.411575	1.903131
67	1	H	-4.319359	3.537344	-1.532339
68	1	H	-4.331997	1.971864	-2.371285
69	1	H	-3.013758	3.132878	-2.680761
70	1	H	-2.502147	3.832406	0.106057
71	1	H	-5.03371	-2.959109	-1.630377
72	1	H	-3.723175	-4.118728	-2.022785
73	1	H	-4.034296	-2.711876	-3.091029
conformation **2**-2c					
Center	Atomic	Atomic	Coordinates (Angstroms)		
Number	Number	Type	X	Y	Z
1	6	C	1.528552	-0.590984	0.905059
2	6	C	2.151037	-0.830058	-0.508654
3	6	C	1.305112	-1.839909	-1.271293
4	6	C	-0.118735	-1.39074	-1.517318
5	6	C	-0.841344	-1.116015	-0.17425
6	6	C	0.026389	-0.175039	0.710208
7	6	C	2.278028	0.574783	1.584794
8	6	C	3.79481	0.392129	1.586919
9	6	C	4.342633	0.151975	0.184944
10	6	C	3.688549	-1.044284	-0.536914
11	6	C	-2.189365	-0.346552	-0.378008
12	6	C	-2.977827	-0.096359	0.967575

13	6	C	-2.017706	0.137151	2.143085
14	6	C	-0.691064	0.111096	2.000961
15	6	C	-1.998632	1.084691	-0.973906
16	6	C	-2.852257	2.120423	-0.238478
17	6	C	-3.663377	1.262859	0.734535
18	6	C	4.168064	-2.335721	0.149415
19	6	C	4.176993	-1.071459	-1.995717
20	8	O	4.080092	1.326058	-0.616682
21	6	C	4.966321	2.330236	-0.574119
22	6	C	4.534136	3.46152	-1.467059
23	8	O	5.974956	2.311686	0.097802
24	6	C	-1.100089	-2.482992	0.498253
25	6	C	-4.029185	-1.153992	1.336439
26	6	C	-3.137439	-1.027324	-1.375519
27	8	O	-3.044401	-2.134011	-1.845606
28	8	O	-4.670139	1.637204	1.289474
29	8	O	-1.286256	1.364034	-1.90699
30	6	C	-2.64494	0.391602	3.490004
31	6	C	1.605628	-1.833486	1.784372
32	6	C	-3.695909	2.97577	-1.16481
33	8	O	-1.918513	2.894881	0.50375
34	8	O	1.706826	-2.923515	-1.653596
35	8	O	-4.139264	-0.187096	-1.664391
36	8	O	1.630314	-1.793252	2.995291
37	1	H	0.121369	0.773624	0.163888
38	1	H	1.97788	0.119983	-1.034582
39	6	C	-5.151342	-0.669044	-2.561654
40	1	H	-0.639348	-2.170163	-2.074262
41	1	H	-0.090437	-0.481381	-2.124043
42	1	H	1.93623	0.690792	2.614928
43	1	H	2.025956	1.498247	1.051161
44	1	H	4.079473	-0.443287	2.234574
45	1	H	4.273587	1.282237	2.005671
46	1	H	5.425721	0.0039	0.21974

47	1	H	-0.08692	0.287283	2.885397
48	1	H	3.698162	-3.20469	-0.313834
49	1	H	5.253624	-2.427931	0.032214
50	1	H	3.961595	-2.364854	1.222988
51	1	H	3.855058	-1.990015	-2.490536
52	1	H	5.27213	-1.034125	-2.023745
53	1	H	3.796553	-0.217373	-2.563013
54	1	H	5.270462	4.263867	-1.42943
55	1	H	4.42552	3.103295	-2.494636
56	1	H	3.559359	3.836841	-1.142644
57	1	H	-0.255608	-3.156955	0.334137
58	1	H	-1.967002	-2.970829	0.0514
59	1	H	-1.260585	-2.393685	1.574714
60	1	H	-4.702448	-1.363687	0.501842
61	1	H	-4.647035	-0.778593	2.154188
62	1	H	-3.561837	-2.087361	1.654466
63	1	H	-1.888025	0.721053	4.206063
64	1	H	-3.427418	1.155582	3.439056
65	1	H	-3.10921	-0.517926	3.887667
66	1	H	1.623486	-2.81042	1.26656
67	1	H	-4.29801	3.669502	-0.570314
68	1	H	-4.369058	2.361995	-1.766994
69	1	H	-3.035978	3.541968	-1.826419
70	1	H	-2.423332	3.510901	1.054445
71	1	H	-4.70656	-0.919437	-3.525798
72	1	H	-5.85993	0.150015	-2.664617
73	1	H	-5.638587	-1.547927	-2.135838
conformation **2**-3c					
Center	Atomic	Atomic	Coordinates (Angstroms)		
Number	Number	Type	X	Y	Z
1	6	C	1.465899	-0.348251	1.026617
2	6	C	2.094268	-0.916592	-0.28722
3	6	C	1.220753	-2.041517	-0.823546
4	6	C	-0.186051	-1.615227	-1.184762

5	6	C	-0.910871	-1.029127	0.053348
6	6	C	-0.017464	0.063389	0.712037
7	6	C	2.251162	0.913936	1.442969
8	6	C	3.75989	0.684215	1.513769
9	6	C	4.31404	0.11983	0.210918
10	6	C	3.623003	-1.183426	-0.240481
11	6	C	-2.231637	-0.284696	-0.334931
12	6	C	-3.025188	0.274336	0.911098
13	6	C	-2.064052	0.767816	2.000506
14	6	C	-0.737582	0.672936	1.883807
15	6	C	-1.992454	0.989962	-1.21086
16	6	C	-2.87612	2.156909	-0.766734
17	6	C	-3.705383	1.543339	0.361696
18	6	C	4.047793	-2.306381	0.722589
19	6	C	4.123788	-1.550209	-1.648322
20	8	O	4.106782	1.095836	-0.835388
21	6	C	5.034897	2.048266	-1.001176
22	6	C	4.659197	2.969643	-2.129816
23	8	O	6.03654	2.138933	-0.324764
24	6	C	-1.201359	-2.196953	1.023811
25	6	C	-4.084974	-0.662898	1.508547
26	6	C	-3.249115	-1.039153	-1.198024
27	8	O	-4.164296	-0.439928	-1.731436
28	8	O	-4.735964	2.0127	0.779521
29	8	O	-1.19592	1.084925	-2.11459
30	6	C	-2.689542	1.361998	3.236594
31	6	C	1.489839	-1.362582	2.164079
32	6	C	-3.709566	2.751502	-1.886292
33	8	O	-2.035943	3.123825	-0.149605
34	8	O	1.588313	-3.194719	-0.952253
35	8	O	-3.077179	-2.344414	-1.295957
36	8	O	1.493322	-1.050101	3.334877
37	1	H	0.123943	0.851329	-0.039354
38	1	H	1.960624	-0.101702	-1.013421

39	6	C	-4.045129	-3.055858	-2.086886
40	1	H	-0.721564	-2.485107	-1.56569
41	1	H	-0.12458	-0.86152	-1.974936
42	1	H	1.903824	1.267613	2.41536
43	1	H	2.039316	1.703566	0.713106
44	1	H	4.005741	0.004482	2.335942
45	1	H	4.267217	1.628158	1.733991
46	1	H	5.390106	-0.054622	0.299047
47	1	H	-0.13429	1.047844	2.704541
48	1	H	3.549221	-3.239501	0.455436
49	1	H	5.129998	-2.460155	0.646298
50	1	H	3.831663	-2.089172	1.772434
51	1	H	3.769643	-2.543004	-1.933367
52	1	H	5.219717	-1.560427	-1.664173
53	1	H	3.78288	-0.829492	-2.396915
54	1	H	3.700263	3.448731	-1.912317
55	1	H	5.430442	3.72846	-2.259106
56	1	H	4.541195	2.396924	-3.053989
57	1	H	-0.380958	-2.918778	1.009626
58	1	H	-2.091945	-2.746538	0.719589
59	1	H	-1.337012	-1.856001	2.052463
60	1	H	-4.752039	-1.057306	0.737953
61	1	H	-4.707621	-0.102552	2.208357
62	1	H	-3.627021	-1.496874	2.042416
63	1	H	-1.92548	1.827941	3.863682
64	1	H	-3.441583	2.118748	2.991135
65	1	H	-3.190787	0.592622	3.834635
66	1	H	1.488344	-2.431256	1.880257
67	1	H	-4.312328	3.56787	-1.481148
68	1	H	-4.366479	1.998517	-2.325394
69	1	H	-3.048218	3.140999	-2.666547
70	1	H	-1.437539	3.457527	-0.834724
71	1	H	-5.039766	-2.937102	-1.654151
72	1	H	-3.730996	-4.096591	-2.052903

73	1	H	-4.037443	-2.681329	-3.111377
conformation **2**-4c					
Center	Atomic	Atomic	Coordinates (Angstroms)		
Number	Number	Type	X	Y	Z
1	6	C	1.526699	-0.604587	0.897803
2	6	C	2.147693	-0.824483	-0.519691
3	6	C	1.300021	-1.82241	-1.296151
4	6	C	-0.123712	-1.368514	-1.534296
5	6	C	-0.844293	-1.111829	-0.186532
6	6	C	0.025153	-0.183741	0.709523
7	6	C	2.27848	0.550531	1.59306
8	6	C	3.794902	0.365	1.591893
9	6	C	4.341662	0.144007	0.186372
10	6	C	3.68487	-1.040273	-0.552542
11	6	C	-2.191873	-0.339514	-0.379564
12	6	C	-2.979575	-0.105112	0.970292
13	6	C	-2.017977	0.111381	2.147948
14	6	C	-0.691459	0.087339	2.003962
15	6	C	-2.006514	1.098727	-0.955844
16	6	C	-2.857545	2.126436	-0.206914
17	6	C	-3.663034	1.259447	0.76176
18	6	C	4.162936	-2.342593	0.114021
19	6	C	4.171653	-1.046789	-2.012135
20	8	O	4.081498	1.330493	-0.597765
21	6	C	4.97106	2.331148	-0.541709
22	6	C	4.541333	3.476959	-1.41719
23	8	O	5.980464	2.299602	0.128492
24	6	C	-1.102384	-2.487509	0.468353
25	6	C	-4.031722	-1.166739	1.324082
26	6	C	-3.141238	-1.00717	-1.384865
27	8	O	-3.051667	-2.1098	-1.864937
28	8	O	-4.68173	1.614017	1.30358
29	8	O	-1.267854	1.404345	-1.862952
30	6	C	-2.643953	0.34797	3.498647

31	6	C	1.602919	-1.858993	1.760228
32	6	C	-3.712998	2.991902	-1.112309
33	8	O	-1.979508	2.899405	0.601675
34	8	O	1.700452	-2.900768	-1.694188
35	8	O	-4.138852	-0.160045	-1.667818
36	8	O	1.625033	-1.83482	2.971585
37	1	H	0.122298	0.771234	0.175158
38	1	H	1.975119	0.133116	-1.031939
39	6	C	-5.1515	-0.629555	-2.571124
40	1	H	-0.645714	-2.139612	-2.101491
41	1	H	-0.095397	-0.450171	-2.127391
42	1	H	1.937342	0.653223	2.624811
43	1	H	2.027945	1.481689	1.072105
44	1	H	4.078117	-0.480188	2.227366
45	1	H	4.275608	1.248002	2.023274
46	1	H	5.424401	-0.006955	0.21857
47	1	H	-0.086381	0.250403	2.890311
48	1	H	3.689667	-3.203561	-0.360733
49	1	H	5.247981	-2.435673	-0.007082
50	1	H	3.958908	-2.386529	1.187573
51	1	H	3.847689	-1.957576	-2.519842
52	1	H	5.266804	-1.010726	-2.040919
53	1	H	3.791846	-0.184006	-2.566562
54	1	H	3.570194	3.853255	-1.083066
55	1	H	5.282184	4.274701	-1.37153
56	1	H	4.426201	3.133751	-2.449147
57	1	H	-0.257774	-3.159003	0.295186
58	1	H	-1.969212	-2.969815	0.015567
59	1	H	-1.262382	-2.412278	1.545958
60	1	H	-4.709119	-1.358328	0.488443
61	1	H	-4.645356	-0.806243	2.151544
62	1	H	-3.565027	-2.107123	1.621668
63	1	H	-1.886192	0.667119	4.218428
64	1	H	-3.425276	1.113772	3.458545

65	1	H	-3.108742	-0.566429	3.884327
66	1	H	1.622695	-2.828842	1.229421
67	1	H	-4.280277	3.693787	-0.496507
68	1	H	-4.40766	2.38628	-1.697972
69	1	H	-3.070099	3.548932	-1.800852
70	1	H	-1.405037	3.39789	0.001444
71	1	H	-4.705852	-0.875063	-3.536105
72	1	H	-5.855288	0.194188	-2.669394
73	1	H	-5.644549	-1.508879	-2.15301
conformation **2**-5c					
Center	Atomic	Atomic	Coordinates (Angstroms)		
Number	Number	Type	X	Y	Z
1	6	C	1.52469	-0.547814	0.916667
2	6	C	2.156477	-0.842294	-0.482765
3	6	C	1.307124	-1.867748	-1.221329
4	6	C	-0.113875	-1.418387	-1.489275
5	6	C	-0.838723	-1.105079	-0.156534
6	6	C	0.028998	-0.127991	0.686402
7	6	C	2.279029	0.635726	1.560215
8	6	C	3.79403	0.439178	1.580804
9	6	C	4.351008	0.143651	0.193226
10	6	C	3.692041	-1.072185	-0.49049
11	6	C	-2.192451	-0.353125	-0.382167
12	6	C	-2.991523	-0.090108	0.954247
13	6	C	-2.029343	0.228174	2.107795
14	6	C	-0.698384	0.235464	1.954735
15	6	C	-2.006622	1.075751	-0.988159
16	6	C	-2.865263	2.112794	-0.252378
17	6	C	-3.714692	1.245075	0.679078
18	6	C	4.153831	-2.342578	0.245305
19	6	C	4.192189	-1.155588	-1.943302
20	8	O	4.105588	1.290048	-0.65209
21	6	C	5.00382	2.284814	-0.642421
22	6	C	4.590962	3.385505	-1.581359

23	8	O	6.00783	2.27982	0.036432
24	6	C	-1.087095	-2.448836	0.563744
25	6	C	-4.007263	-1.162838	1.367918
26	6	C	-3.131443	-1.056752	-1.369739
27	8	O	-3.03075	-2.172974	-1.814996
28	8	O	-4.764928	1.580052	1.17098
29	8	O	-1.281286	1.348285	-1.914321
30	6	C	-2.653996	0.52341	3.447861
31	6	C	1.58062	-1.755659	1.846014
32	6	C	-3.673992	2.980804	-1.191573
33	8	O	-2.030338	2.973677	0.513089
34	8	O	1.70372	-2.964535	-1.568651
35	8	O	-4.136744	-0.227459	-1.67479
36	8	O	1.562494	-1.663772	3.054031
37	1	H	0.146382	0.784387	0.085921
38	1	H	1.999111	0.091297	-1.042234
39	6	C	-5.148656	-0.731417	-2.560573
40	1	H	-0.634636	-2.211232	-2.026439
41	1	H	-0.081585	-0.524769	-2.119242
42	1	H	1.932557	0.790223	2.583709
43	1	H	2.039393	1.542247	0.992763
44	1	H	4.065514	-0.374792	2.260744
45	1	H	4.2773	1.33951	1.971411
46	1	H	5.432298	-0.012647	0.24305
47	1	H	-0.098653	0.478788	2.826168
48	1	H	3.675844	-3.223011	-0.187322
49	1	H	5.238593	-2.451478	0.135842
50	1	H	3.943589	-2.329399	1.318518
51	1	H	3.868733	-2.089505	-2.40727
52	1	H	5.287695	-1.125894	-1.96281
53	1	H	3.822298	-0.320336	-2.544576
54	1	H	3.616822	3.781917	-1.281091
55	1	H	5.334741	4.181691	-1.566964
56	1	H	4.488776	2.989507	-2.595676

57	1	H	-0.242255	-3.125889	0.416546
58	1	H	-1.955517	-2.953522	0.138721
59	1	H	-1.240596	-2.327163	1.638322
60	1	H	-4.661423	-1.436589	0.536524
61	1	H	-4.648797	-0.766203	2.157079
62	1	H	-3.51601	-2.063351	1.738543
63	1	H	-1.908278	0.92254	4.139327
64	1	H	-3.474978	1.242897	3.36927
65	1	H	-3.066975	-0.390467	3.889045
66	1	H	1.62274	-2.752523	1.369879
67	1	H	-4.285908	3.667369	-0.601821
68	1	H	-4.325037	2.371318	-1.820975
69	1	H	-2.992108	3.551149	-1.82693
70	1	H	-1.557505	2.446955	1.177429
71	1	H	-4.704572	-0.996179	-3.521165
72	1	H	-5.861985	0.081734	-2.675899
73	1	H	-5.629243	-1.605385	-2.117547
conformation **2**-6c					
Center	Atomic	Atomic	Coordinates (Angstroms)		
Number	Number	Type	X	Y	Z
1	6	C	1.461828	-0.301575	1.029076
2	6	C	2.099768	-0.92383	-0.255724
3	6	C	1.225748	-2.063829	-0.759645
4	6	C	-0.178129	-1.646909	-1.146637
5	6	C	-0.907754	-1.021486	0.068609
6	6	C	-0.016031	0.099478	0.678797
7	6	C	2.247917	0.974186	1.401577
8	6	C	3.7551	0.741809	1.494443
9	6	C	4.318651	0.123338	0.22056
10	6	C	3.626972	-1.194763	-0.184564
11	6	C	-2.23346	-0.298184	-0.342291
12	6	C	-3.037943	0.274807	0.888752
13	6	C	-2.076761	0.84062	1.942
14	6	C	-0.74489	0.775534	1.810768

15	6	C	-1.98975	0.968186	-1.233921
16	6	C	-2.869159	2.142519	-0.787711
17	6	C	-3.744812	1.512705	0.297506
18	6	C	4.039627	-2.280304	0.825389
19	6	C	4.139189	-1.617618	-1.57252
20	8	O	4.124023	1.056918	-0.86566
21	6	C	5.059322	1.996277	-1.064409
22	6	C	4.697047	2.871763	-2.2331
23	8	O	6.056564	2.10882	-0.384899
24	6	C	-1.191365	-2.15369	1.081345
25	6	C	-4.071539	-0.659884	1.529444
26	6	C	-3.241988	-1.073924	-1.193413
27	8	O	-4.155491	-0.487971	-1.743955
28	8	O	-4.801903	1.955314	0.677218
29	8	O	-1.211085	1.03072	-2.153826
30	6	C	-2.703465	1.467735	3.161474
31	6	C	1.468839	-1.266188	2.210238
32	6	C	-3.654057	2.751986	-1.929222
33	8	O	-2.060936	3.16903	-0.225508
34	8	O	1.589829	-3.221906	-0.843846
35	8	O	-3.067114	-2.380816	-1.256059
36	8	O	1.430349	-0.899669	3.364465
37	1	H	0.142328	0.840873	-0.11514
38	1	H	1.978333	-0.136655	-1.013982
39	6	C	-4.029331	-3.11516	-2.033438
40	1	H	-0.712259	-2.528148	-1.502272
41	1	H	-0.112533	-0.918288	-1.960007
42	1	H	1.895265	1.366033	2.357298
43	1	H	2.045035	1.735672	0.639901
44	1	H	3.991151	0.095516	2.345953
45	1	H	4.263641	1.692299	1.680773
46	1	H	5.393168	-0.050932	0.325898
47	1	H	-0.145462	1.209622	2.604813
48	1	H	3.536522	-3.220609	0.593985

49	1	H	5.121068	-2.443999	0.760405
50	1	H	3.820679	-2.019109	1.864629
51	1	H	3.788473	-2.621305	-1.82091
52	1	H	5.235184	-1.62738	-1.578344
53	1	H	3.80472	-0.927575	-2.352239
54	1	H	3.737845	3.362339	-2.044493
55	1	H	5.472217	3.622044	-2.386511
56	1	H	4.585365	2.26172	-3.133903
57	1	H	-0.367368	-2.871113	1.095164
58	1	H	-2.078249	-2.718926	0.794602
59	1	H	-1.331165	-1.778806	2.097793
60	1	H	-4.727665	-1.103144	0.776155
61	1	H	-4.70849	-0.08271	2.20245
62	1	H	-3.597315	-1.458966	2.100222
63	1	H	-1.952244	2.001204	3.74852
64	1	H	-3.501559	2.169236	2.898911
65	1	H	-3.1485	0.699098	3.803007
66	1	H	1.494881	-2.346147	1.975633
67	1	H	-4.284376	3.553906	-1.537372
68	1	H	-4.283115	1.998622	-2.406244
69	1	H	-2.956263	3.160556	-2.664277
70	1	H	-1.574414	2.808444	0.532878
71	1	H	-5.026528	-2.987038	-1.609406
72	1	H	-3.713269	-4.153904	-1.970314
73	1	H	-4.016399	-2.76754	-3.067304

4. Spectra of 1

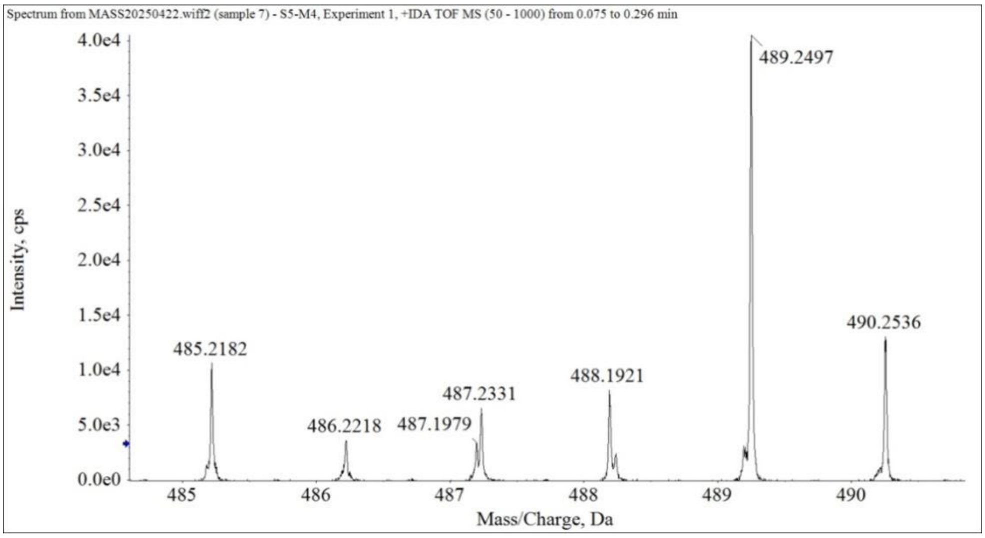

Hit	Formula	m/z	RDB	ppm	MS Rank	MSMS ppm	MSMS Rank	Found
1	C27H34O8	487.2326	11.0	0.9	1			NA/NA

Figure S5. HRESIMS spectrum of **1**.

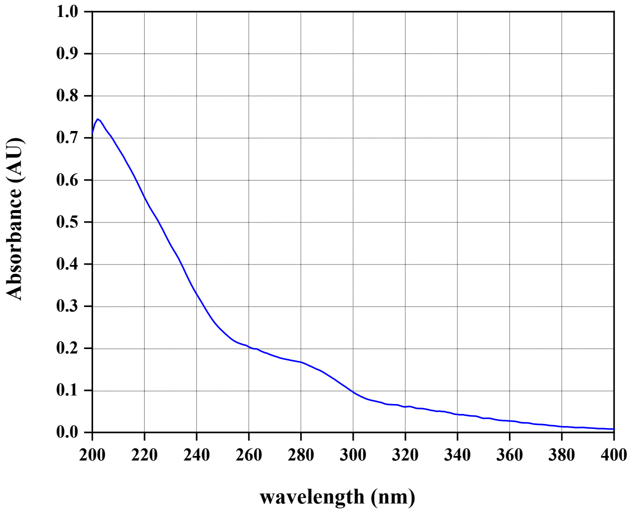

Figure S6. UV spectrum of **1** (MeOH).

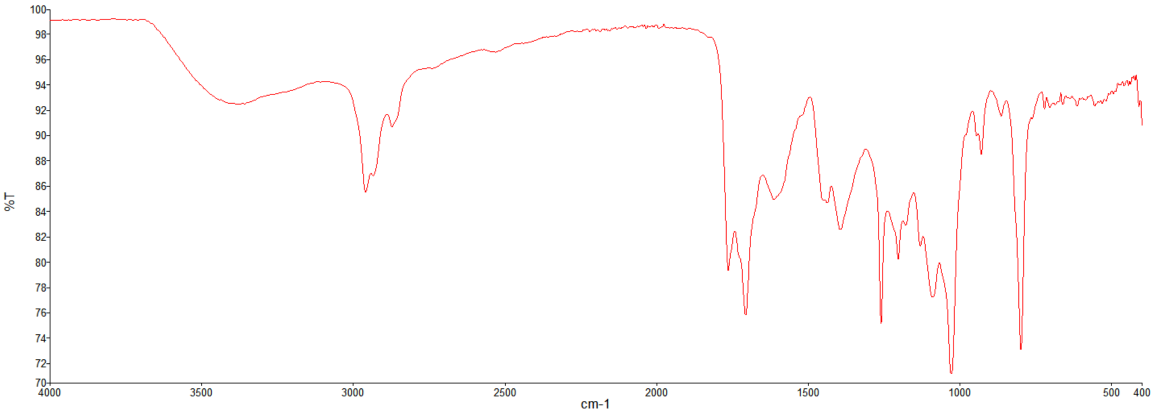

Figure S7. IR spectrum of **1** (MeOH).

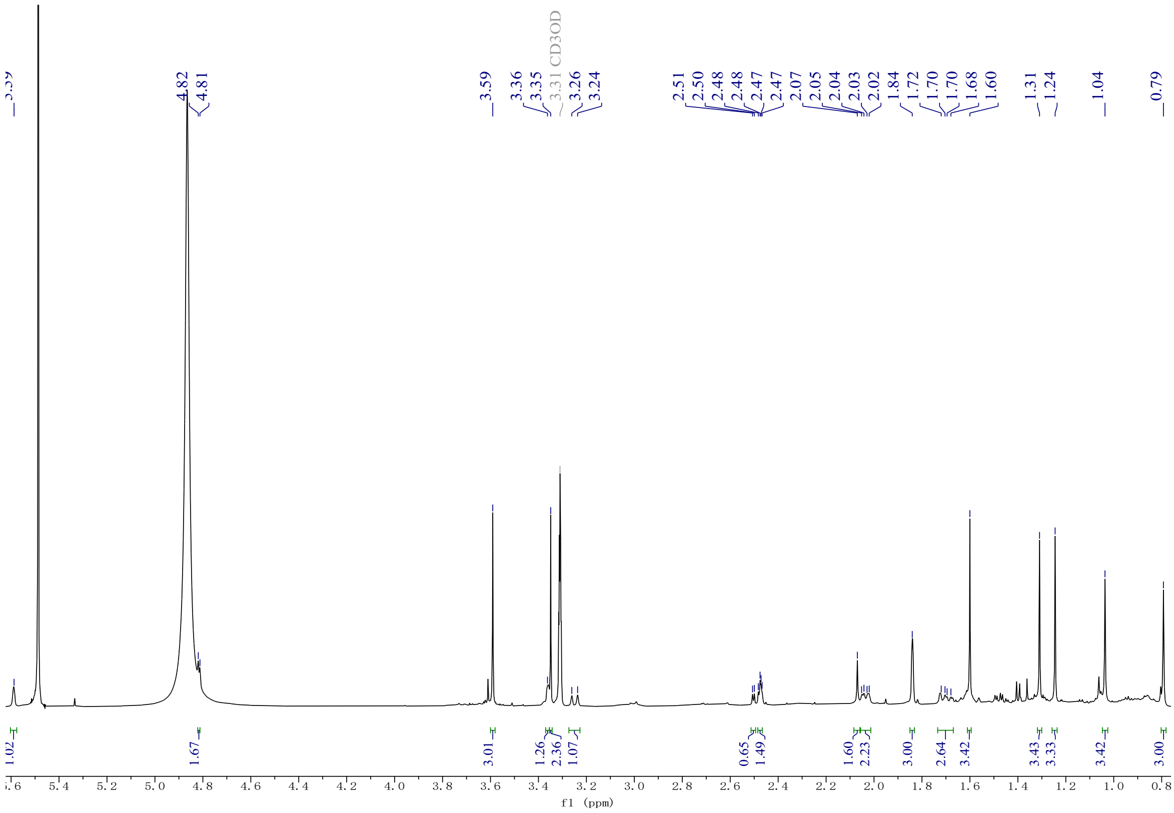

Figure S8. ^{1}H NMR spectrum (600 MHz, CD_3OD) of **1**.

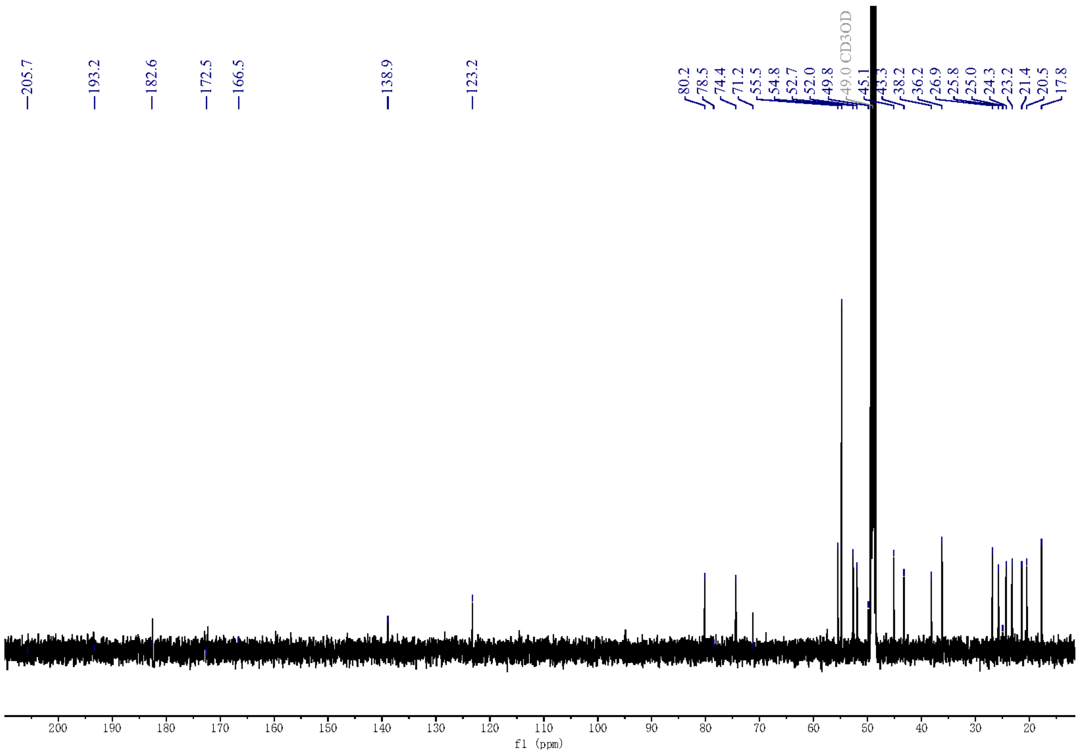

Figure S9. ^{13}C NMR spectrum (150 MHz, CD_3OD) of **1**.

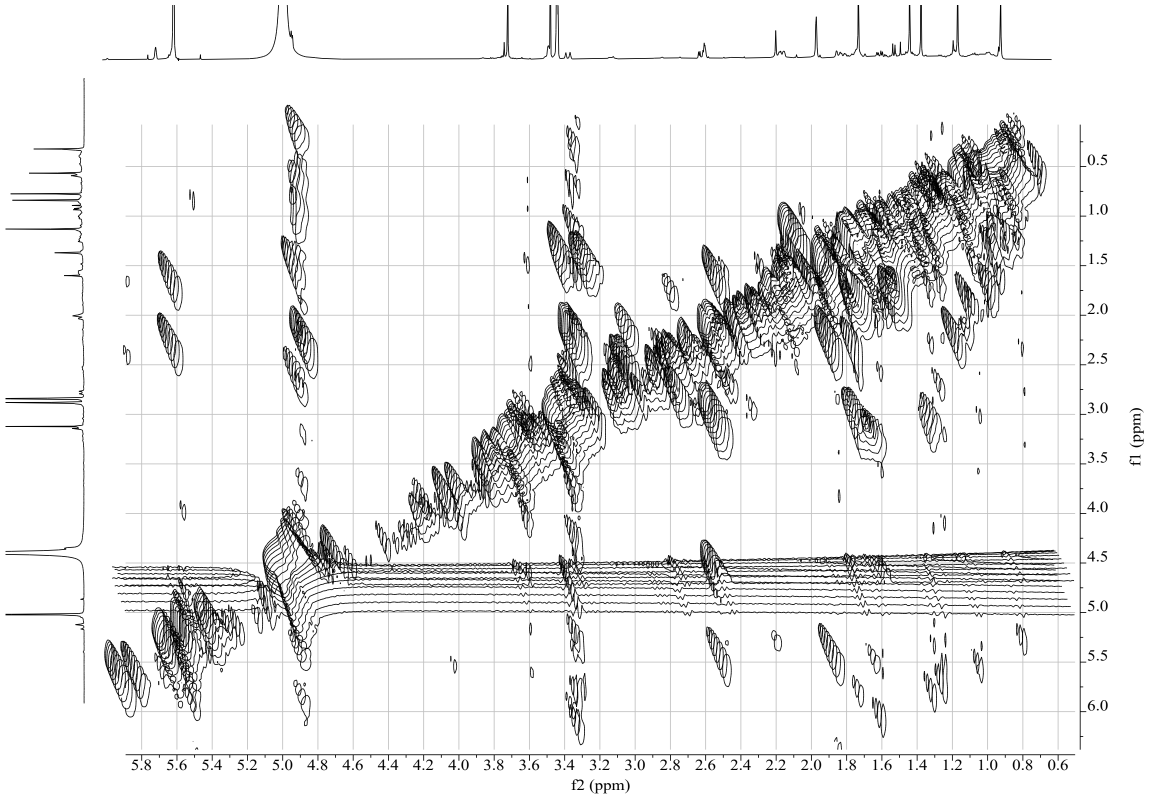

Figure S10. ^{1}H-^{1}H-COSY spectrum of **1**.

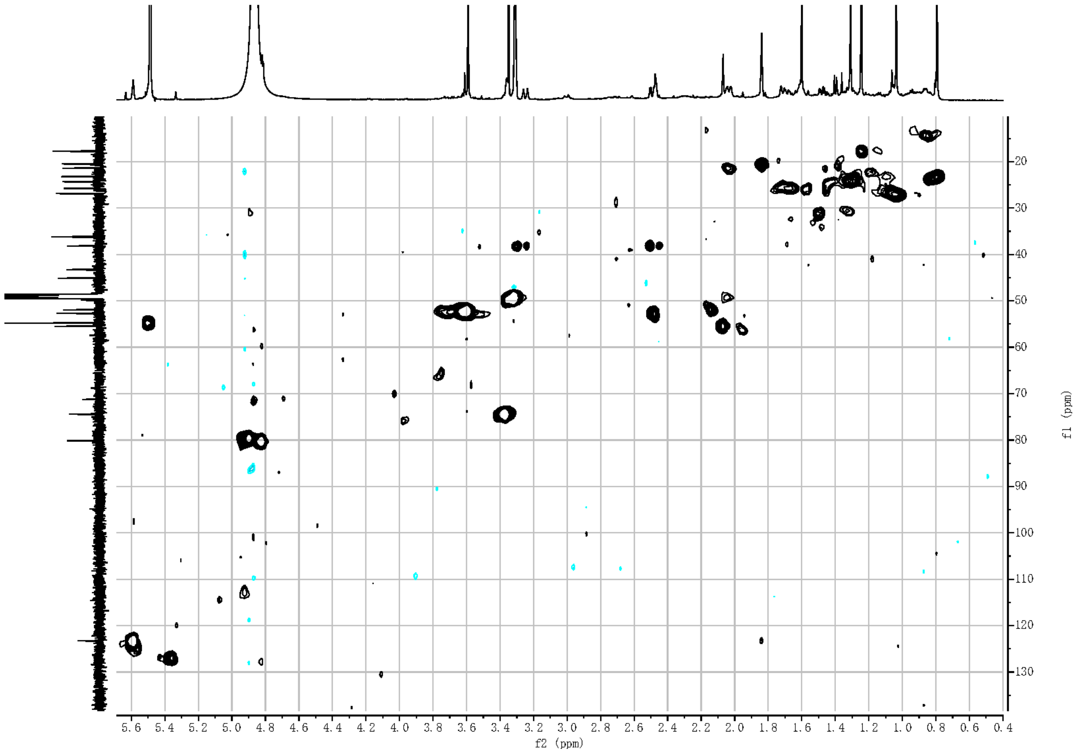

Figure S11. HSQC spectrum of **1**.

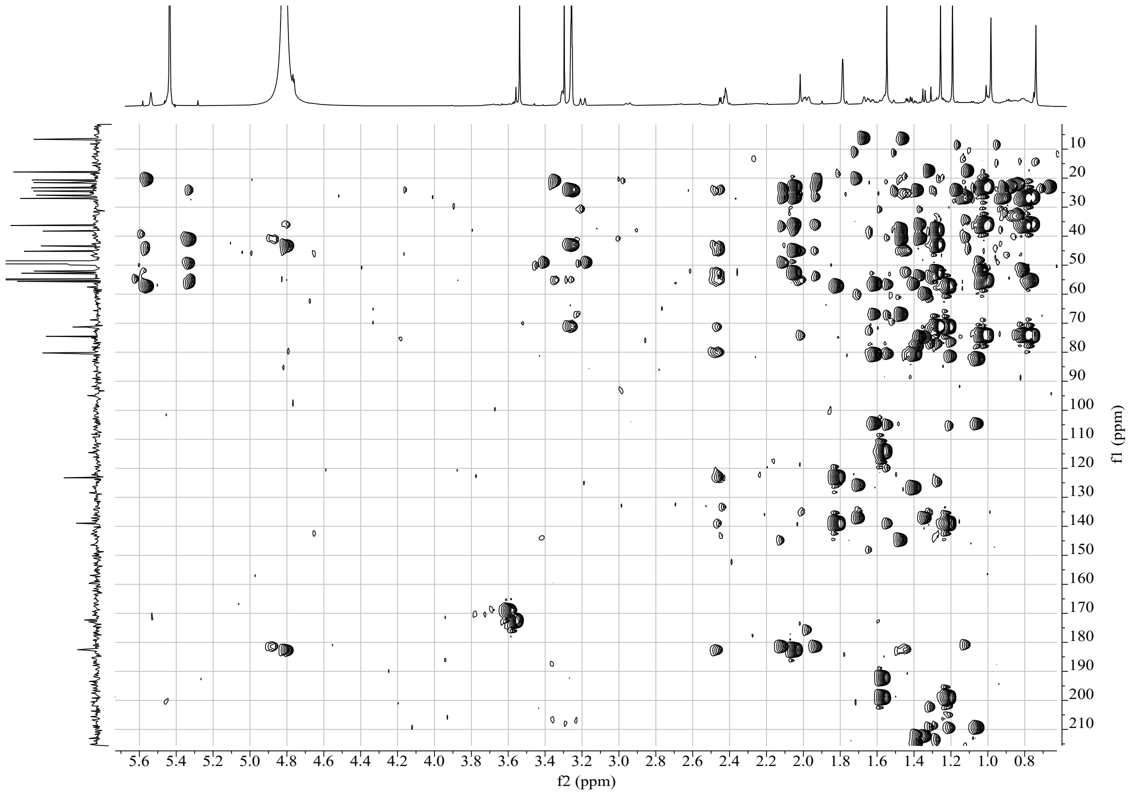

Figure S12. HMBC spectrum of **1**.

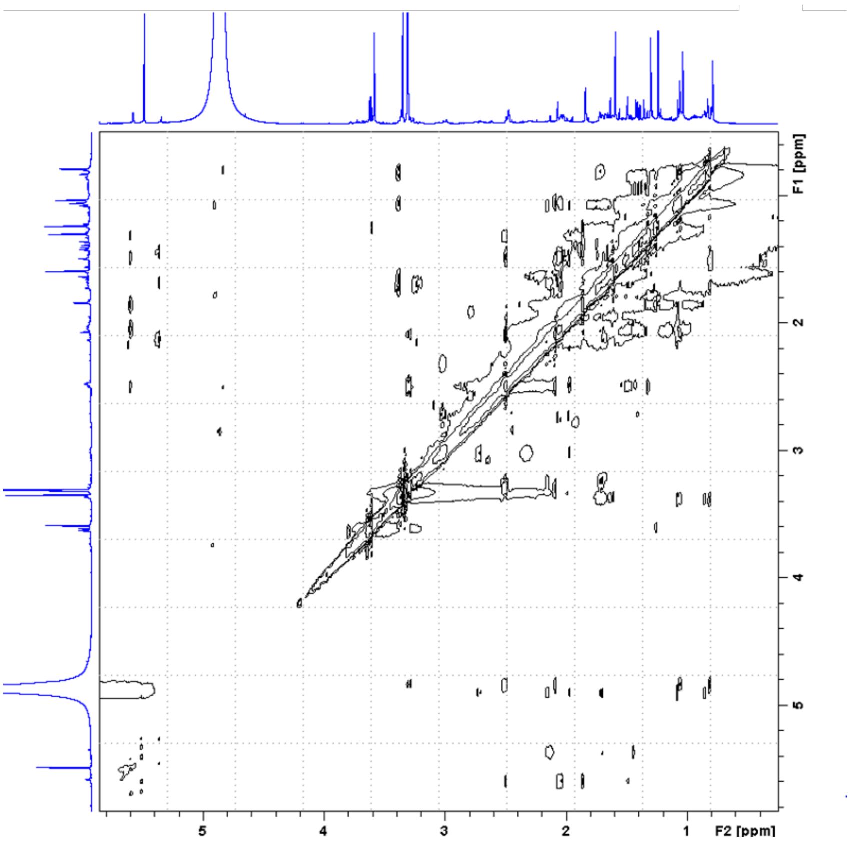

Figure S13. NOESY spectrum of **1**.

5. Spectra of 2

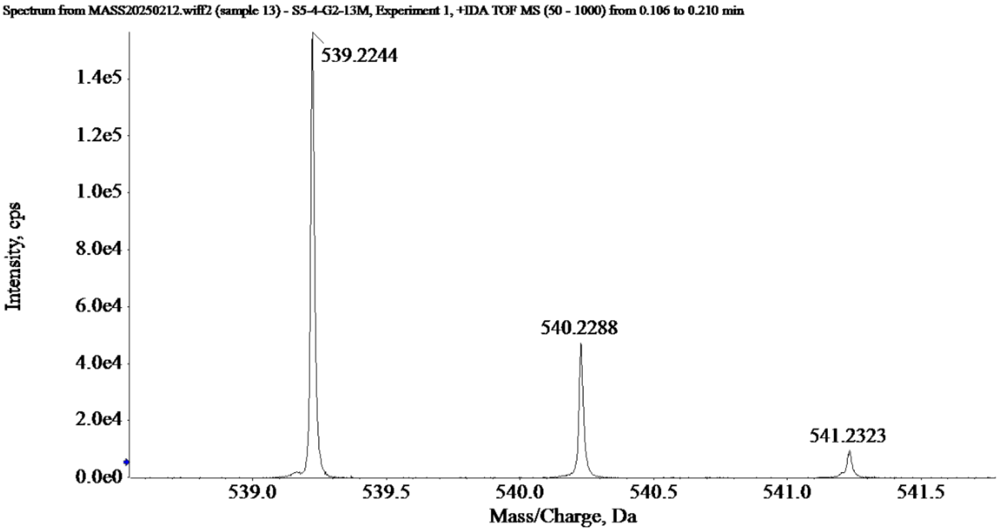

Hit	Formula	m/z	RDB	ppm	MS Rank	MSMS ppm	MSMS Rank	Found
1	C28H36O9	539.2252	11.0	-1.4	1			NA/NA

Figure S14. HRESIMS spectrum of **2**.

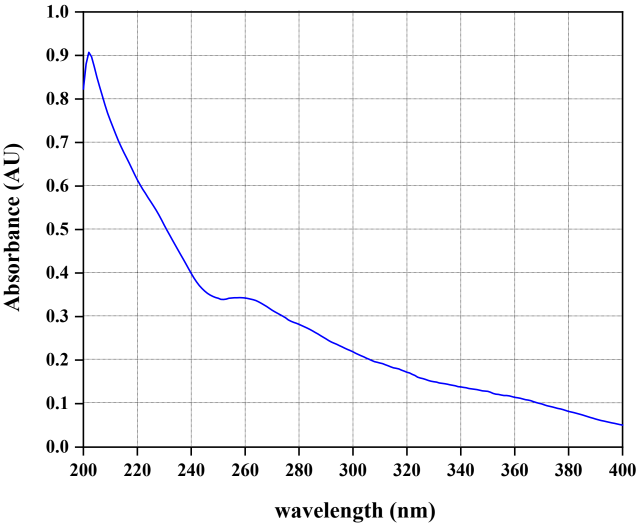

Figure S15. UV spectrum of **2** (MeOH).

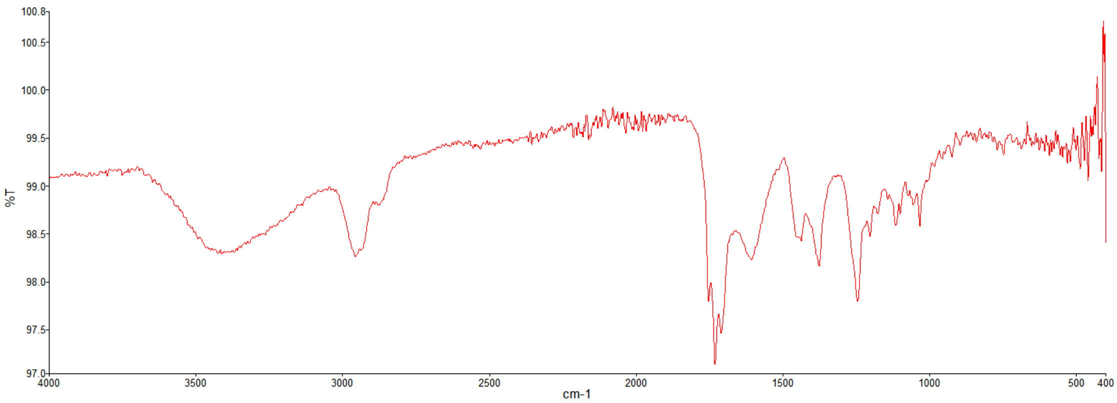

Figure S16. IR spectrum of **2** (MeOH).

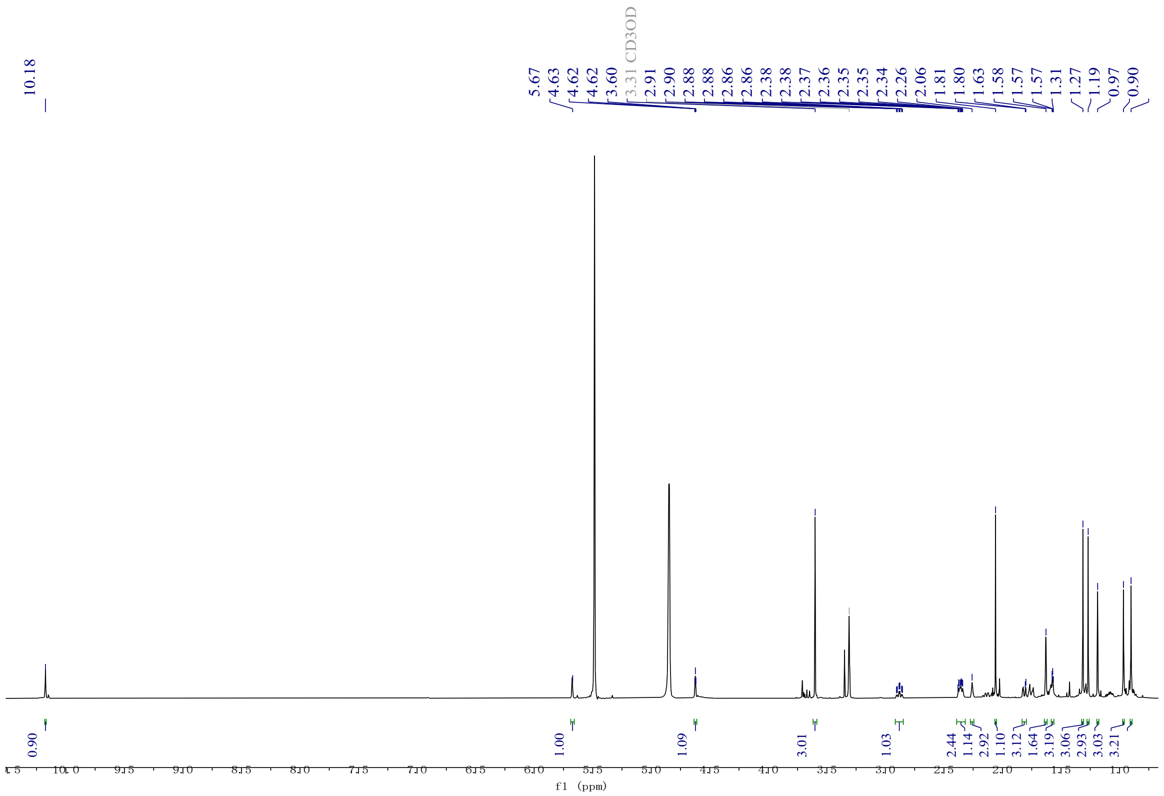

Figure S17. ^{1}H NMR spectrum (600 MHz, CD_3OD) of **2**.

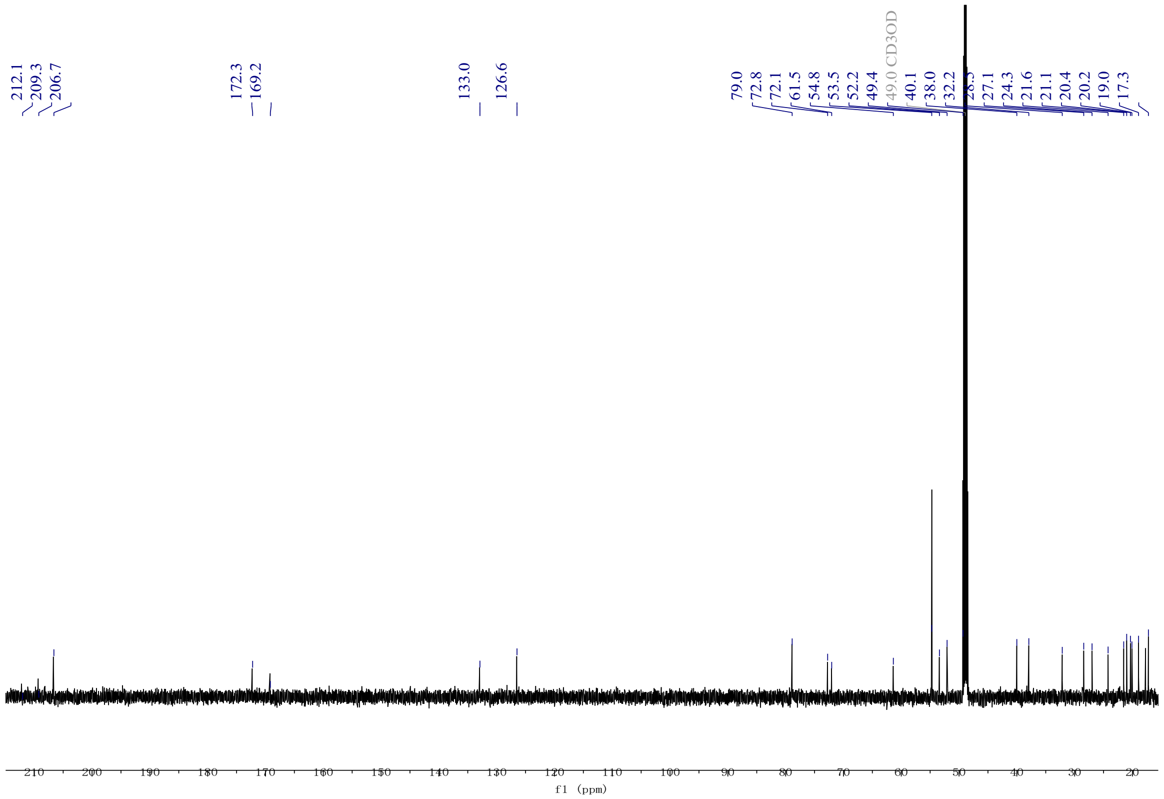

Figure S18. ^{13}C NMR spectrum (150 MHz, CD_3OD) of **2**.

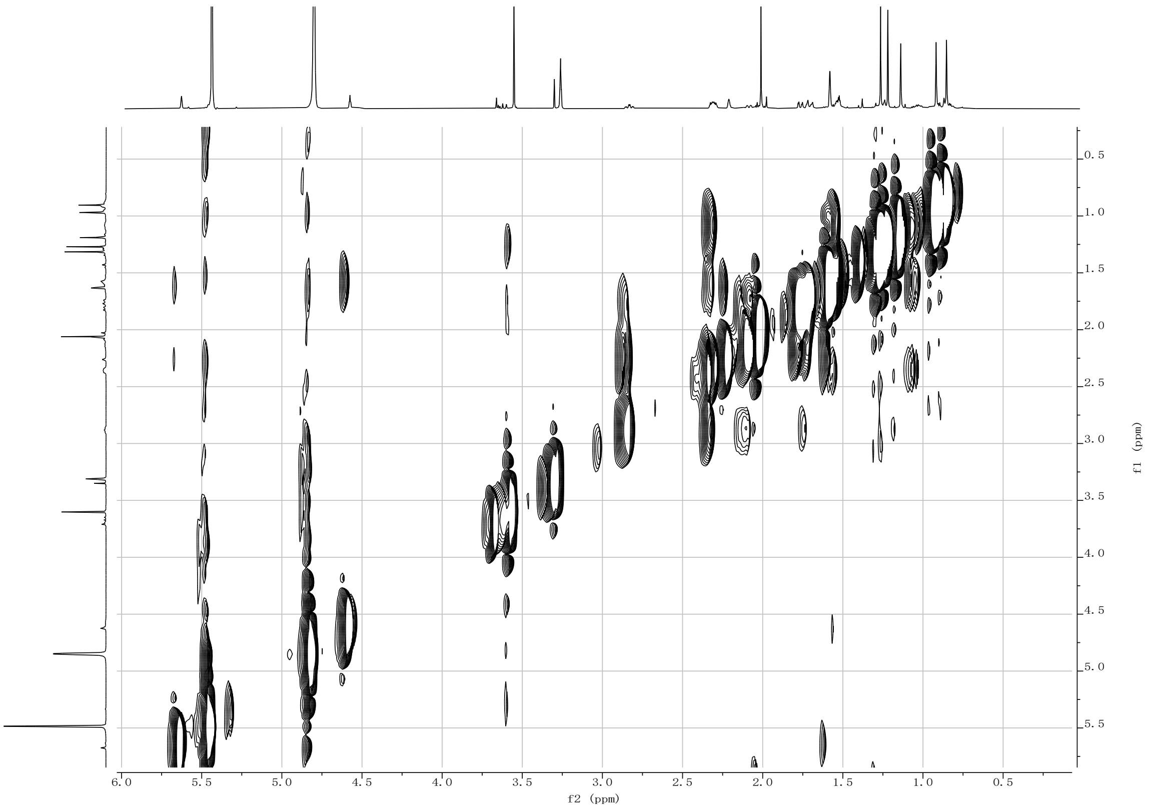

Figure S19. ^{1}H-^{1}H-COSY spectrumof **2**.

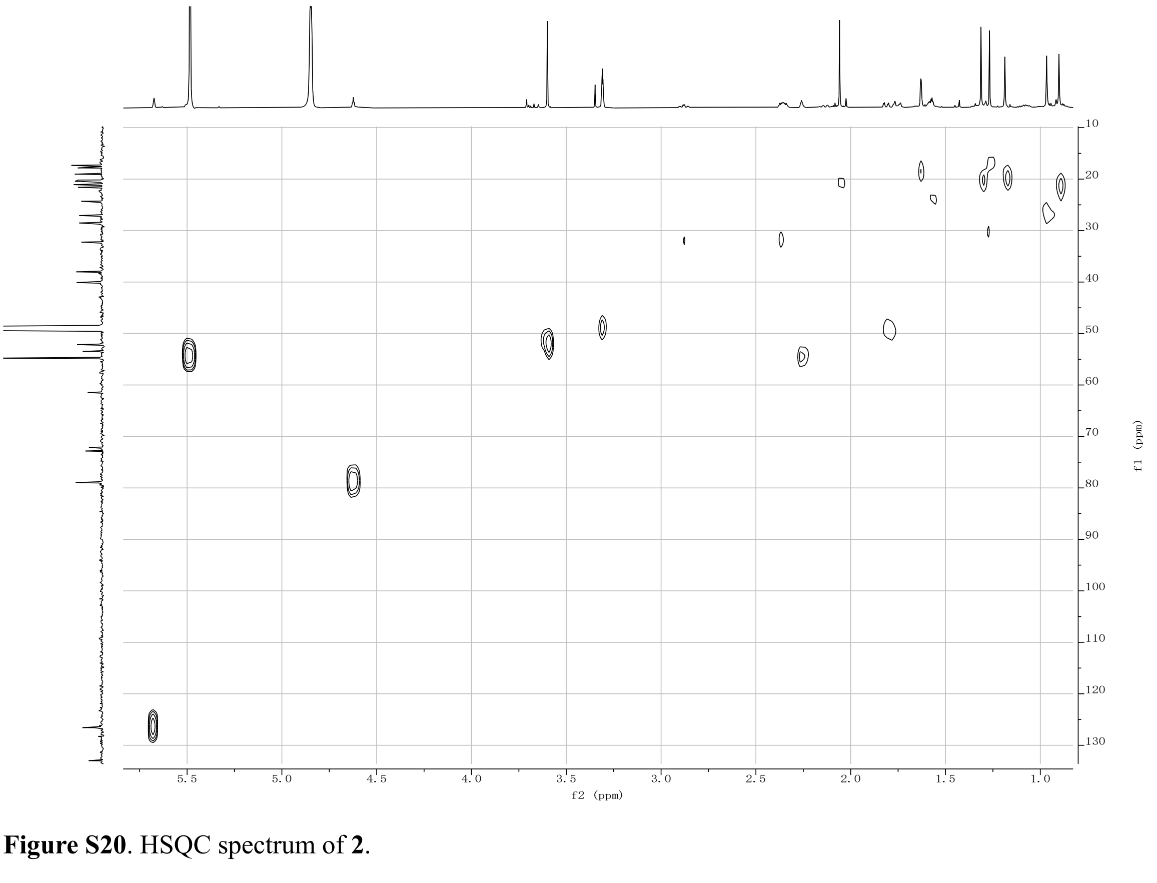

Figure S20. HSQC spectrum of **2**.

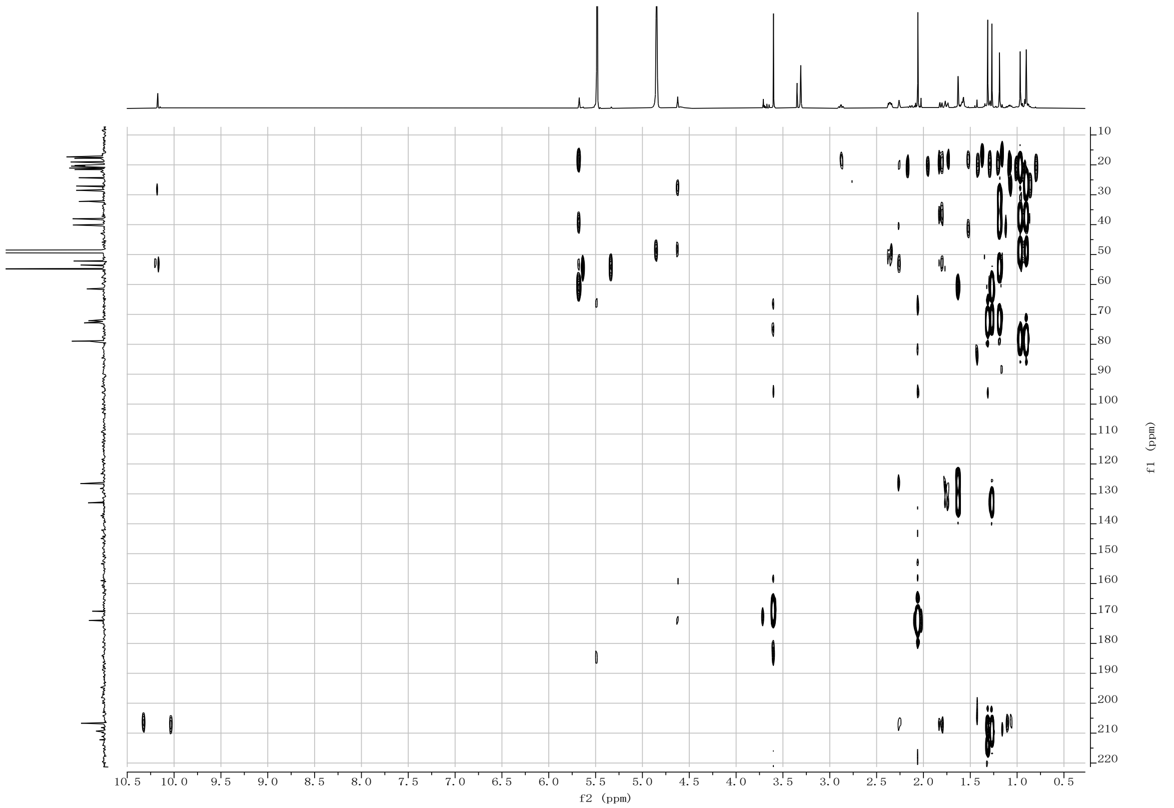

Figure S21. HMBC spectrum of **2**.

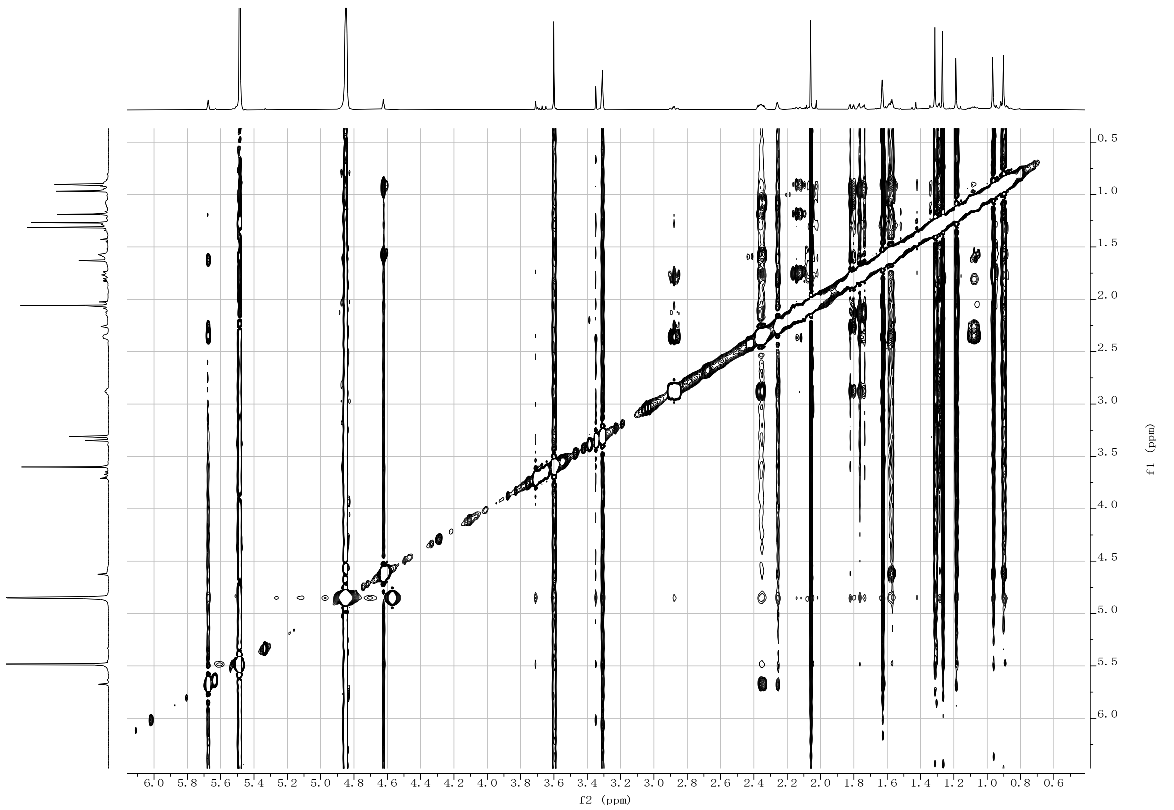

Figure S22. NOESY spectrum of **2**.
